# Ecofriendly, Highly Selective Lithium Extraction by Redox-Mediated Electrodialysis

**DOI:** 10.1021/acscentsci.4c01373

**Published:** 2024-11-09

**Authors:** Rongxuan Xie, Danyi Sun, Jinyao Tang, Xiaochen Shen, Parsa Pishva, Yanlin Zhu, Kevin Huang, Zhenmeng Peng

**Affiliations:** † Department of Chemical Engineering, University of South Carolina, Columbia, South Carolina 29208, United States; ‡ Department of Mechanical Engineering, University of South Carolina, Columbia, South Carolina 29208, United States

## Abstract

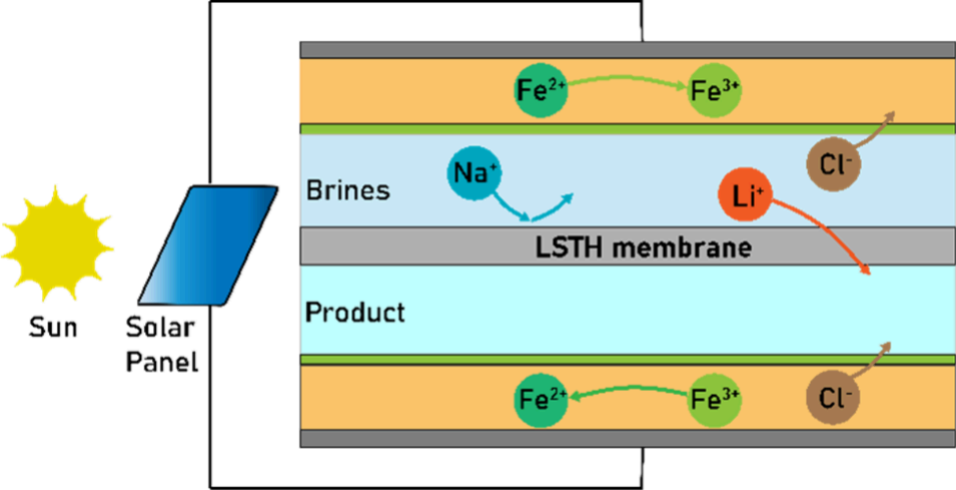

The rapid proliferation of lithium battery applications has underscored the critical role of lithium supply in the transition to industrial electrification. Existing lithium production methods encounter significant challenges in efficiency, scalability, environmental impact, and cost. The integration of redox-mediated electrodialysis with a dense ceramic Li_6/16_Sr_7/16_Ta_3/4_Hf_1/4_O_3_ perovskite membrane, distinguished by its unique lattice structure allowing only lithium-ion exchange and transport, enables efficient, highly lithium-selective extraction directly from a diversity of resources including seawater and various brines. This approach offers continuous operation capability, can utilize renewable power, and has notable advantages, including chemical-free operation and little waste generation. Overall, this innovative solution presents a one-step, ecofriendly, highly selective lithium extraction method.

## Abstract 2

The one-step redox-mediated electrodialysis (rm-ED) strategy for direct lithium extraction from brines with LSTH membrane is employed for high selectivity powered by solar panels.

## Introduction

**Figure 1:**
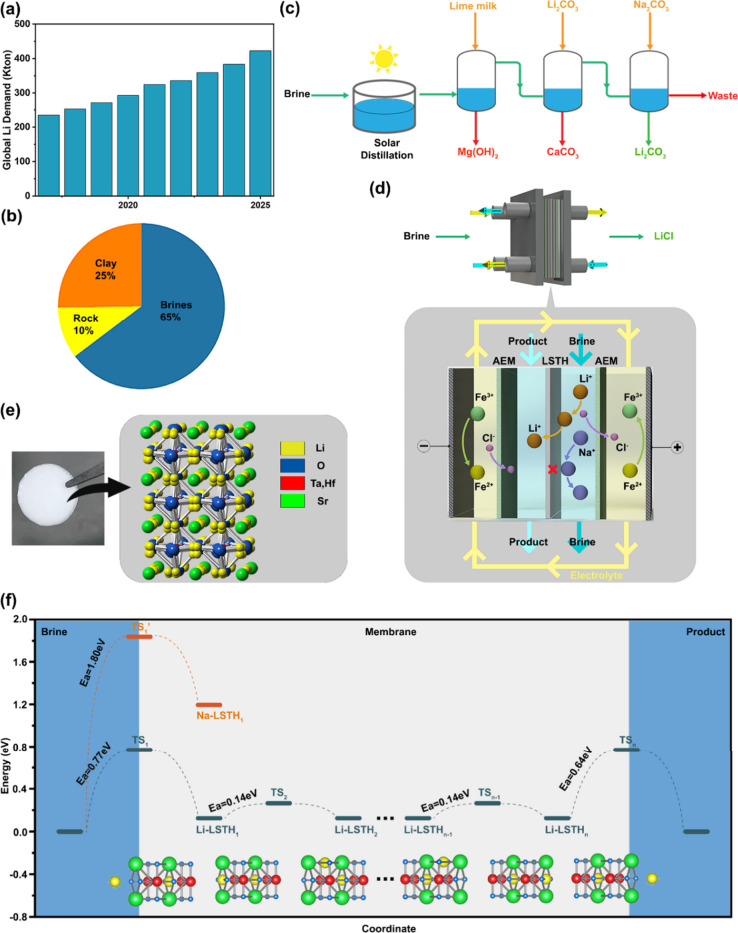
(a) Global lithium demand; (b) Distribution of lithium reserves; (c) State-of-the-art multistage lithium extraction process; (d) Illustration of the one-step redox-mediated electrodialysis (rm-ED) strategy for direct lithium extraction from diverse brines, with (e) Li_6/16_Sr_7/16_Ta_3/4_Hf_1/4_O_3_ (LSTH) perovskite membrane employed for high selectivity; and (f) DFT simulation of Li-ion exchange and transport pathway through LSTH.

Lithium, one of the most valuable metals in the world, has been widely used in various industries as a strategic resource.^1−3^ Its significance has surged in recent years, driven by the expansive utilization of lithium-ion batteries. Projections indicate that global lithium consumption will soar to 422 kilotons by 2025 (Figure 1a).^4^ Consequently, the price of lithium has increased rapidly from $6,000 per ton in 2016 to nearly $60,000 per ton in 2022, with no signs of abating.^5^ This escalating demand underscores the critical need to acquire lithium from diverse sources.^6−8^

The majority of lithium reserves are found in various types of brine water (Figure 1b). More than 50% of global lithium production is sourced from continental brine through an extensive multistage process encompassing evaporation, purification, and extraction (Figure 1c).^9−11^ This process requires prolonged operation duration (usually 2–3 years), a large land footprint, and heavy chemical usage, impacting the economic viability and raising environmental concerns. Furthermore, its limited effectiveness in processing brine with low lithium concentrations renders this process nearly nonviable for extracting lithium from abundant sources like seawater.^12,13^ While seawater represents the most readily available lithium resource, its lithium concentration is exceedingly low, typically ranging from 0.1 to 0.2 ppm. Moreover, the presence of high levels of competing ions, including Na^+^, K^+^, Mg^2+^, and Ca^2+^, further complicates the extraction process.^5,14^ Different lithium extraction approaches have emerged in response to these challenges, such as nanofiltration,^15^ solvent extraction,^16^ ion sieve adsorption,^17^ and others. While these alternative methods hold promise for enhancing lithium recovery and accessing lower concentration sources, they encounter significant hurdles related to selectivity, scalability, versatility, environmental sustainability, and cost-effectiveness. Therefore, the quest for discovering and developing an advanced lithium extraction method remains imperative.

Herein, we report a redox-mediated electrodialysis (rm-ED) method designed for energy-efficient, continuous, direct extraction of lithium, with purity exceeding 99%, from various brine sources. This method employs a dense ceramic Li_6/16_Sr_7/16_Ta_3/4_Hf_1/4_O_3_ (LSTH) perovskite membrane that exclusively permits lithium-ion exchange at the interface and facilitates transport through its lattice structure, achieving exceptional lithium selectivity. Moreover, it does not consume any chemicals, generates no waste, and can be powered by renewable energy, ensuring ecofriendliness.

## Results and Discussion

Figures 1d and S1–S3 illustrate the rm-ED cell system for study in this work. It comprises two anion exchange membranes (AEMs) and one LSTH membrane sandwiched between two graphite end plate electrodes, thereby dividing the cell into four channels. The outmost two channels are circulated with a Fe^2+^/Fe^3+^ redox electrolyte, while the middle two channels carry a brine stream (i.e., feed) and a lithium extraction stream (i.e., product), respectively. Applying a cell voltage induces electrochemical redox reactions at the electrodes (Figure S4), resulting in an accumulation of excess negative charges in the catholyte and excess positive charges in the anolyte adjacent to the electrodes. This charge distribution creates an electrostatic field within the cell, facilitating the directed migration of cations from the brine stream to the extraction stream through the LSTH membrane, along with the migration of anions from the brine stream to the anolyte and from the catholyte to the extraction stream through the AEMs. AEMs effectively prevent cations from migrating into the Fe^2+^/Fe^3+^ redox electrolyte, thereby preventing contamination and ensuring the stability of the entire rm-ED system (Figure S5). The LSTH membrane’s lattice specificity for sole lithium-ion exchange restricts the passage of all other ions (Figures 1e and S6), allowing only lithium ions to permeate into the extraction stream while retaining other ions in the brine stream.^18^ As a result, lithium salt is extracted from the brine and concentrated in the product channel. Through the circulation of electrolytes, the redox chemicals are consumed and replenished simultaneously. This dynamic process establishes an equilibrium in their concentrations, sustaining a stable electrostatic field and thus ensuring a continuous lithium extraction process.

The LSTH membrane was synthesized via a solid-state reaction method reported in our previous work.^19^ It is characterized as A-site deficient Li_6/16_Sr_7/16_Ta_3/4_Hf_1/4_O_3_ in perovskite lattice structure and exhibits a promising bulk ionic conductivity of 0.408 mS·cm^–1^ (Figure S7, Table S1). The membrane possesses a smooth microstructural surface consisting of well-defined grains with intact grain boundaries (Figure S8). It shows excellent stability in both acidic and neutral environments, as evidenced by minimal changes in appearance, composition, and structure after an extended testing (Figures S9 and S10). Density functional theory (DFT) simulations indicate that lithium ions are preferentially exchanged into the LSTH interface lattice from solution due to their significantly lower energy barrier (*E*_a_ = 0.77 eV) compared to most other cations like sodium ions (*E*_a_ = 1.80 eV), leading to exceptional lithium selectivity. Additionally, a low 0.14 eV energy barrier for lithium ions to hop between adjacent sites in the LSTH lattice suggests their high mobility, ensuring the effective transport of lithium ions across the membrane (Figure 1f).

The impact of key operational parameters, including lithium-ion concentration in the brine stream and cell voltage, and of competing cations on the rm-ED lithium extraction properties were examined. The lithium-ion concentration in the brine stream exhibits significant influences on its extraction rate as well as the energy consumption (Figures S11 and 2a,b). A lithium extraction rate of 88 mmol·h^–1^·m^–2^ and an energy consumption of 0.23 kWh·mol^–1^ were achieved when a 10 mM LiCl brine stream was fed to the rm-ED cell. An increase in the brine concentration leads to an acceleration of the extraction rate and, meanwhile, less energy consumption, reaching a significantly higher 320 mmol·h^–1^·m^–2^ and a lower 0.14 kWh·mol^–1^ with 200 mM LiCl, respectively. With the charge efficiency increasing as the brine concentration increases (Figure S11), this can be attributed to a higher likelihood of lithium ions engaging with the LSTH membrane interface as the brine concentration rises, which promotes better ion exchange kinetics and facilitates greater permeation flux across the membrane. Consequently, the accelerated ion exchange and transport augment both the extraction rate and energy efficiency.

**Figure 2:**
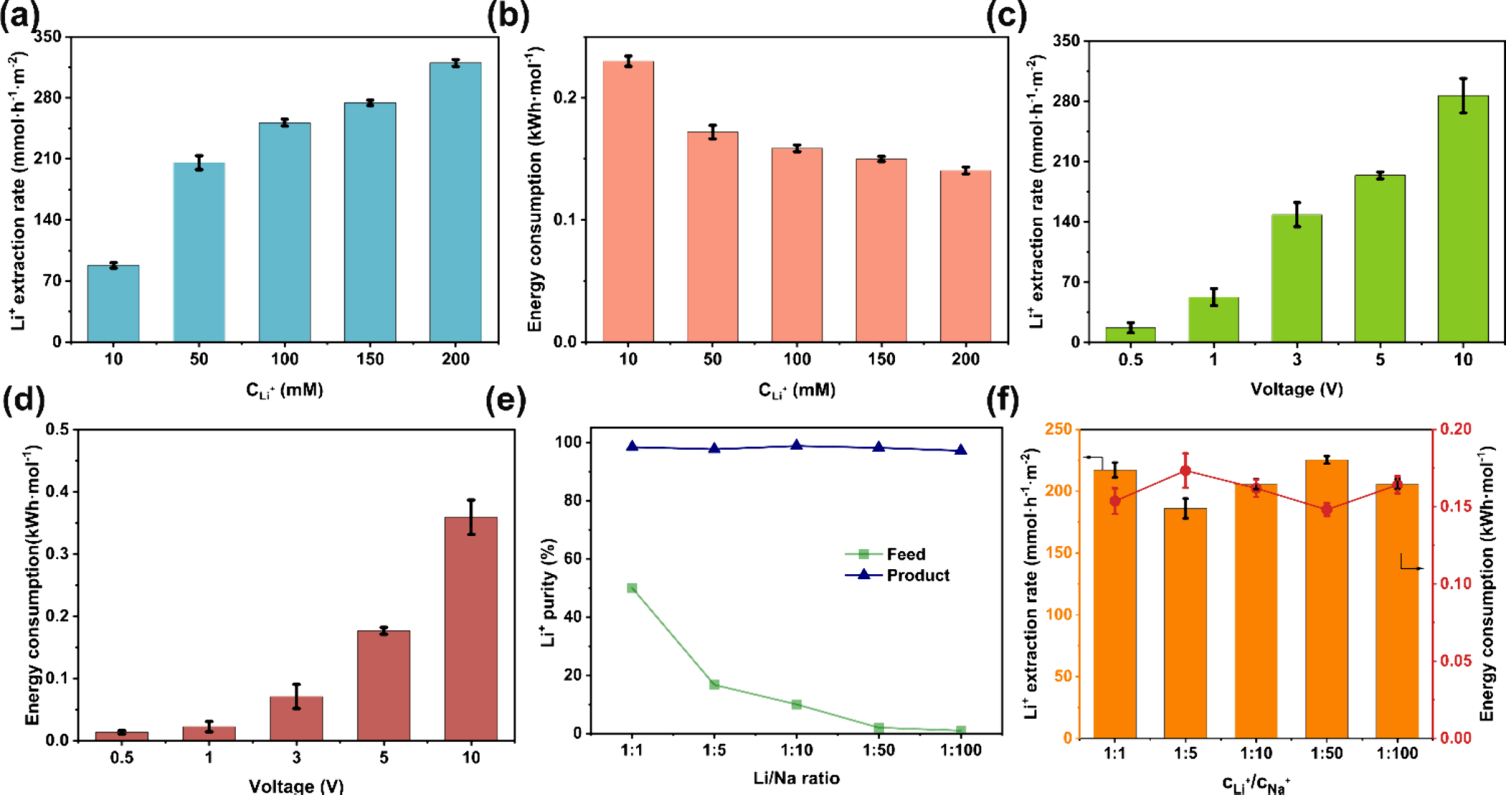
(a, b) Effects of Li-ion concentration in feed on average lithium extraction rate and energy consumption, with 5 V rm-ED cell voltage; (c, d) Effects of applied voltage on average rate and energy consumption for lithium extraction from 50 mM Li-ion feed; (e, f) Effects of Li^+^/Na^+^ ratio in feed on lithium purity in product, average lithium extraction rate, and energy consumption, with Li-ion concentration in feed fixed at 50 mM and 5 V rm-ED cell voltage.

The voltage of the rm-ED cell was found to have a direct correlation with both the lithium extraction rate and the energy consumption (Figures S13, S14, and 2c,d). An extraction rate of only 17.1 mmol·h^–1^·m^–2^ was obtained with a 0.5 V voltage. The rate surged dramatically to 287 mmol·h^–1^·m^–2^ with a 10 V voltage, showcasing a notable enhancement effect. This observation aligns with the understanding that the charge imbalance prompted by the applied cell voltage acts as the primary impetus for ion migration. A higher voltage accelerates the Fe^2+^/Fe^3+^ redox rate, intensifying the generated charge imbalance. Consequently, a stronger electric field is established across the cell, which in turn enhances the lithium-ion extraction process.^20^ Meanwhile, as the cell voltage increased, so did the energy consumption, with values ranging from 0.014 kWh·mol^–1^ at 0.5 V to 0.40 kWh·mol^–1^ at 10 V. The heightened energy consumption can be attributed to the elevated cell current that induces a more pronounced ohmic loss and a gradual decrease in the charge efficiency (Figure S15), resulting in reduced energy utilization efficiency. Hence, a trade-off exists between the lithium extraction rate and energy consumption, which can be managed by regulating the cell voltage. As the cell resistance is further reduced, the lithium extraction rate will be increased with less energy consumption.

The study on the impact of competing cations in the brine stream confirms the remarkable selectivity of the rm-ED cell for lithium extraction. The rm-ED cell was tested using a mix solution containing 50 mM LiCl and various NaCl concentrations. A higher NaCl concentration implies a greater presence of competing Na^+^ ions, increasing the likelihood of contact with and permeation through the LSTH membrane if the latter is not a sole Li-ion conductor. Nevertheless, despite the presence of varying concentrations of competing Na^+^ ions, the extracted lithium consistently achieved over 99% purity in the final product, with minimal sodium product quantified by inductively coupled plasma optical emission spectrometry (ICP-OES) analyses (Figures 2e and S16). The rm-ED cell performance remained remarkably stable, with the lithium extraction rate, energy consumption, and charge efficiency consistently hovering around 200 mmol·h^–1^·m^2^, 0.17 kWh·mol^–1^, and 90%, respectively (Figures 2f, S17, and S18). To further assess the selectivity of the LSTH membrane against competing cations, we tested three mixed solutions containing 50 mM LiCl and 50 mM of either KCl, CaCl_2_, or MgCl_2_. The results demonstrated that the concentrations of the competing cations remained at extremely low levels in the products, indicating high selectivity for Li^+^ (Figures S19 and S20). This observation confirmed the exceptional selectivity of the rm-ED cell for lithium extraction, a characteristic largely attributed to the lithium-ion transport property of the LSTH membrane (Table S2). The membrane’s ability to selectively allow lithium ions to pass through while effectively blocking other ions underscores its crucial role in ensuring high-purity lithium extraction.

**Figure 3:**
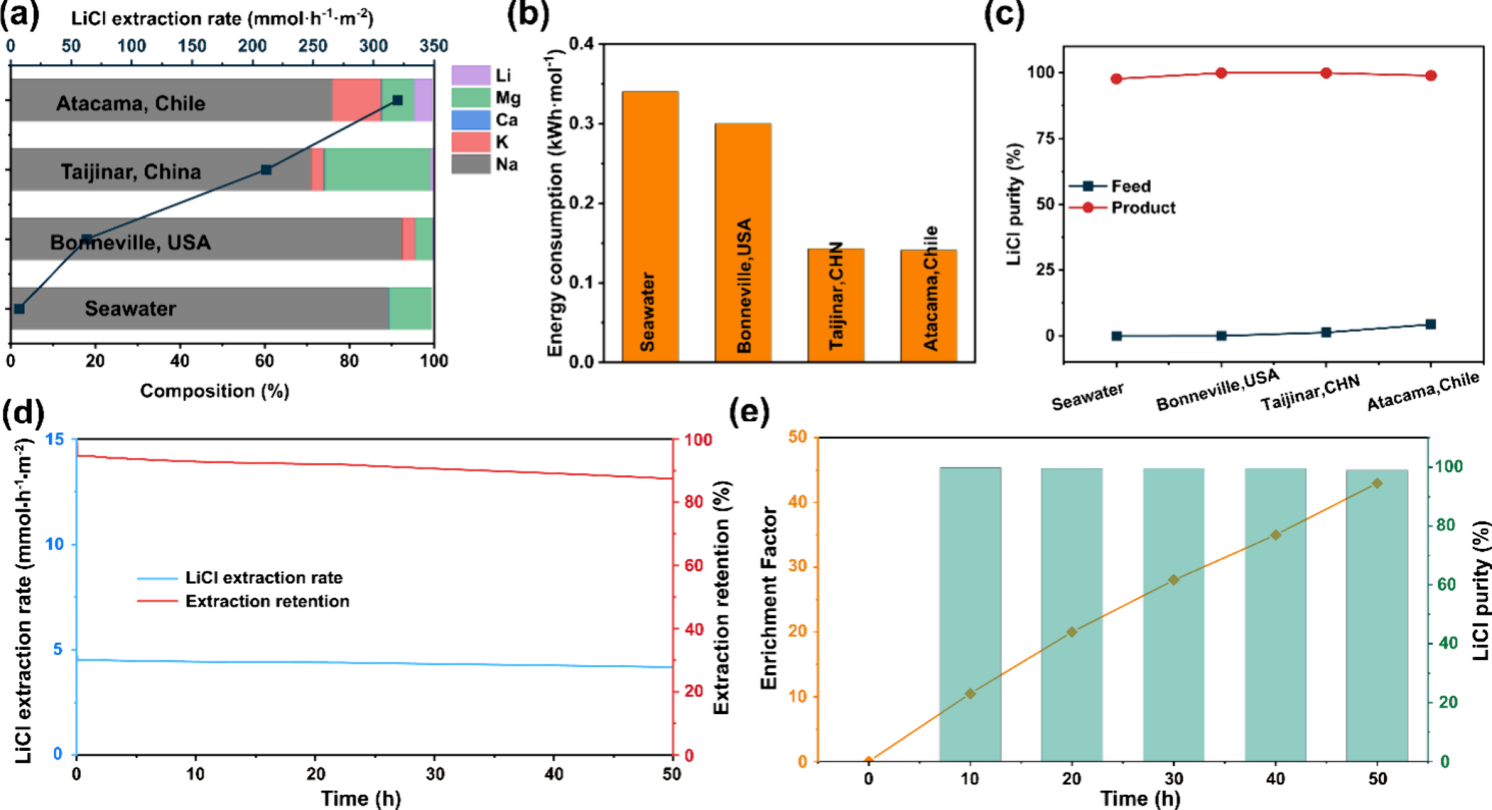
(a, b) Average rate and energy consumption for lithium extraction (converted to LiCl) from simulated brines with different composition and 5 V rm-ED cell voltage, and (c) lithium purity in feed and product; (d) Lithium extraction rate (converted to LiCl) and the rate retention as a function of time with simulated seawater feed and 5 V rm-ED voltage, and (e) lithium enrichment factor and purity in the product.

The rm-ED system’s ability to process real brine containing diverse lithium concentrations and competing ions was assessed using four simulated brines with different compositions (Table S3). At a cell voltage of 5 V, the lithium extraction rates were measured as 320 mmol·h^–1^·m^–2^ for Chile Atacama brine, 211 mmol·h^–1^·m^–2^ for CHN Taijinar, 63 mmol·h^–1^·m^–2^ for USA Bonneville, and 7 mmol·h^–1^·m^–2^ for seawater (Figures 3a, S21, and S22). Conversely, the energy consumption followed the exact opposite order, with extracting lithium from seawater consuming the most energy at 0.34 kWh·mol^–1^ and the least from Chile Atacama, at 0.14 kWh·mol^–1^ (Figure 3b). The trends in lithium extraction rate and energy consumption aligned with the varying lithium concentrations in these simulated brines, consistent with the observed effect of lithium concentration. Across all four simulated brines, the purity of lithium in the product consistently remained close to 100%, underscoring the high selectivity of the LSTH membrane against various cations (Figure 3c).

A key advantage of this rm-ED system is its potential for theoretically infinite extraction capacity with low energy consumption, enabled by continuous operation. This was evaluated through prolonged extraction of lithium from simulated seawater at a 5 V cell voltage (Figure S23). The charge efficiency stabilized at approximately 60% throughout the experiment (Figure S24), indicating excellent system durability. The instantaneous lithium extraction rate remained steady at 6 mmol·h^–1^·m^–2^ initially, gradually declining to 83% of the initial value over 50 h of operation (Figure 3d), likely due to a decrease in lithium concentration in the brine stream. Concurrently, the concentration of lithium in the product steadily increased during the operation, eventually reaching 43 times the initial lithium concentration in the simulated seawater (Figure 3e). Importantly, the lithium purity consistently remained near 100% throughout the operation, affirming the exceptional selectivity and stability of the process.

**Figure 4:**
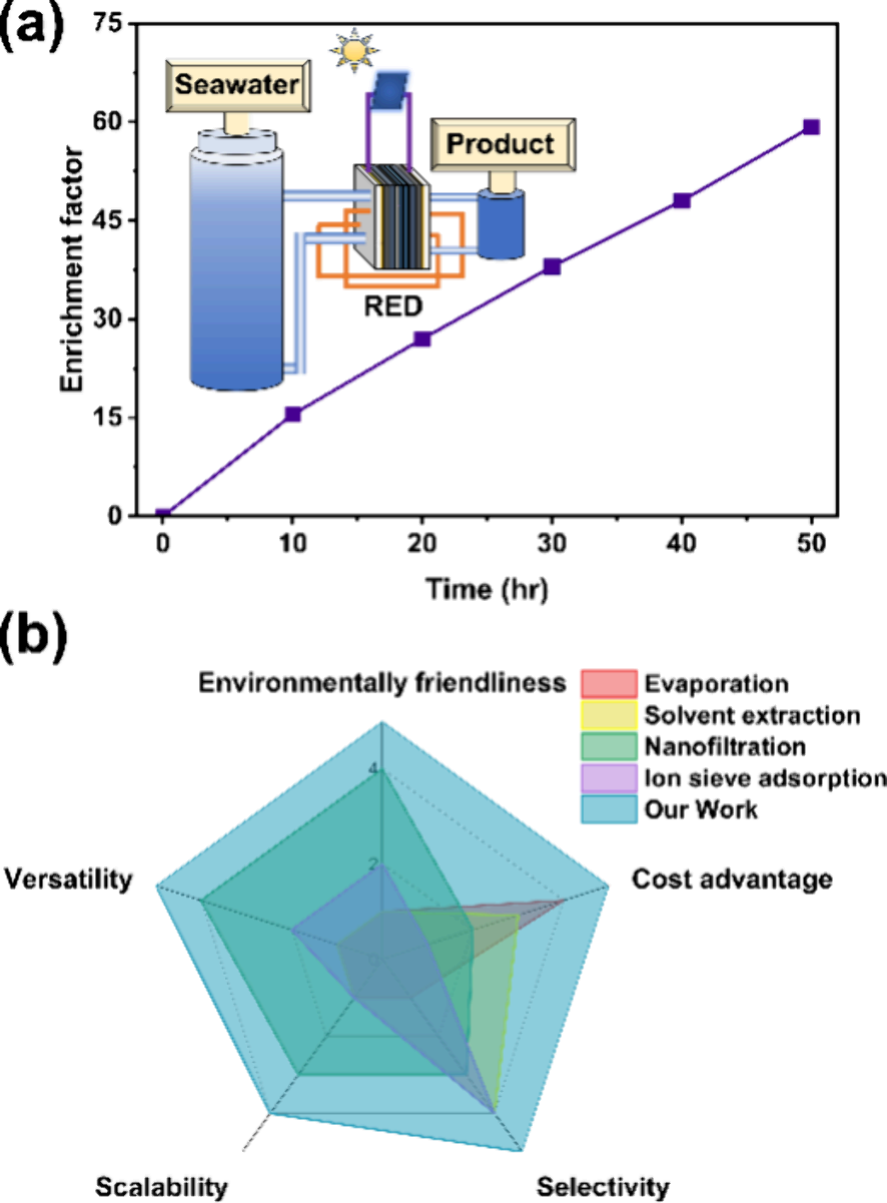
(a) Lithium enrichment factor from simulated seawater with continuous extraction powered by a 6 V solar panel, and (b) comparison of the literature reported separation methods with this work.

The rm-ED system’s low voltage requirements and energy-saving features enable integration with renewable energy sources, facilitating a clean and environmentally friendly lithium extraction process. Demonstrated with a commercial 6.0 V solar panel, the system achieved a lithium extraction rate of 6.8 mmol·h^–1^·m^2^, reaching 59 times the initial concentration of lithium in simulated seawater with exceptional purity after 50 h of operation (Figures 4a and S25). This highlights the potential for using solar or other renewable energy sources to further reduce the cost of lithium production.

Compared to other lithium production methods currently adopted or under active investigation, such as evaporation, solvent extraction, nanofiltration, and ion sieve adsorption, our rm-ED system exhibits superiority across several critical factors (Figure 4b).^15−17,21^ First, it offers environmental friendliness by consuming no chemicals and generating no waste, besides the ability to utilize renewable energy sources. Second, it boasts cost-effectiveness due to its low energy requirements, efficient operation, and no need for postextraction separation. Third, its high selectivity ensures pure lithium extraction, minimizing impurities. Additionally, the scalability and versatility of the rm-ED system make it adaptable to various operational scales and conditions. These advantages underscore the considerable potential of the rm-ED approach for widespread application in lithium production.

## Conclusion

In summary, the use of a dense ceramic Li_6/16_Sr_7/16_Ta_3/4_Hf_1/4_O_3_ (LSTH) perovskite membrane in a redox-mediated electrodialysis (rm-ED) system has been demonstrated to enable clean, continuous, and highly selective lithium extraction from diverse brines. The exceptional selectivity stems from the sole lithium-ion transport property of LSTH that allows only lithium-ion exchange and repels the passage of all other ions. An increase of lithium-ion concentration in the brine was found to accelerate the extraction rate to as high as 320 mmol·h^–1^·m^–2^ and reduce energy consumption to as low as 0.14 kWh·mmol^–1^, while an increase in the rm-ED cell voltage boosts the extraction rate at the expense of more energy consumption. Durable, efficient extraction of lithium with >99% purity has been achieved with a variety of brine compositions and concentrations. The feasibility of using solar to power lithium extraction from seawater has also been demonstrated, showcasing the potential of the new technology to leverage renewable energy for cost reduction and environmental friendliness. With notable advantages in selectivity, cost-effectiveness, scalability, versatility, and environmental sustainability, the LSTH-based rm-ED process promises to be an appealing option for lithium extraction.

## Supporting Information Available

Extended figures detailing rm-ED cell design and setup, along with data from CV, EIS, SEM, and XRD characterizations; chronoamperometry, CE, and concentration profile results from the extraction experiments (PDF)
Transparent Peer Review report available (PDF)
