# Supplementary material for: Ecofriendly, Highly Selective Lithium Extraction by Redox-Mediated Electrodialysis
Source: ACS Cent Sci. 2024 Nov 9;10(11):2119–24. doi: 10.1021/acscentsci.4c01373 (PMC11613207; doi:10.1021/acscentsci.4c01373)
Supplement: Supplementary file 1 — oc4c01373_si_001.pdf [file oc4c01373_si_001.pdf]

Supplementary Information for

Eco-friendly, Highly Selective Lithium Extraction by Redox-mediated Electrodialysis

Rongxuan Xie[1, §], Danyi Sun[1, §], Jinyao Tang[1], Xiaochen Shen[1], Parsa Pishva[1], Yanlin Zhu[1], Kevin Huang[2, *], Zhenmeng Peng[1, *]

[1] Department of Chemical Engineering, University of South Carolina, Columbia, SC, 29208, USA

[2] Department of Mechanical Engineering, University of South Carolina, Columbia, SC, 29208, USA

Email: zmpeng@sc.edu (Z. P.*), huang46@cec.sc.edu (K. H.*), [§] Equal contribution

Materials

Iron (II) chloride (Sigma Aldrich, ≥99%), iron (III) chloride hexahydrate (Sigma Aldrich, ≥99%), lithium chloride (Sigma Aldrich, ≥99%), lithium carbonate (Alfa Aesar, 99.998%), strontium carbonate (Aldrich Chemistry, 99.9%), hafnium(IV) oxide (Alfa Aesar, 99.9%), tantalum pentoxide (Alfa Aesar, 99 993%), hydrochloric acid (Sigma Aldrich, 37%), calcium chloride (Fisher Scientific), potassium chloride (Fisher Scientific), magnesium chloride (Fisher Scientific), and sodium chloride (Fisher Scientific) were used without purification after purchase. All the ion exchange members used in this study were purchased from Fuel Cell Store (College Station, Texas).

Preparation of LSTH Membranes

LSTH was synthesized via the solid-state reaction method, which is described in our previous work[1]. Stoichiometric amounts of high-purity Li_2CO_3, $SrCO_3$, HfO_2, and Ta_2O_5 were intimately mixed and ground using a mortar and pestle in ethanol. The mixed powder was pelletized under 200 MPa by cold isostatic pressing and then calcined at 900°C for 6 hours in the air using a zirconia crucible. Approximately three times more Mother Powder Bed (MPB) powder than LSTH was placed on top of the LSTH pellets and between the LSTH and the zirconia crucible. After pre-calcination, a micronizing mill (McCrone Microscopes & Accessories) was used to reduce the particle size of the sample. The sample was then pressed into a pellet under 200 MPa and sintered at 1350°C.

Characterization

To acquire the crystal structure data, the as-synthesized LSTH pellets were evaluated using powder X-ray diffraction (XRD) with a Rigaku D/MAX-2100. The scans were performed in the 2θ range of 20°–80° with a scanning interval of 0.02°. Ionic conductivity data were obtained using electrochemical impedance spectroscopy (EIS) with a Solartron 1255 frequency response analyzer. Measurements were taken within the frequency range of 1 MHz to 100 Hz, with a 10 mV AC perturbation, across a temperature range of 25 to 72. 4 °C. Prior to each measurement, the two surfaces of an LSTH pellet sample (dimensions: 5.5 mm in radius and 0.87 mm in height) were coated with gold paste and attached to silver wires as leads. To reveal the grain boundaries in the samples, the as-synthesized samples were polished with sandpaper down to 1 μm and annealed at 1100 °C for 2 hours. The samples were sputtered with a thin layer of gold (120 seconds) before imaging to enhance the image quality. A scanning electron microscope (SEM), specifically a Zeiss Ultra Plus, was used to capture the microstructures.

Chemical Stability Tests

The chemical stability of the LSTH membrane in acidic environment was evaluated by placing one LSTH pullet in 1 L of 0.1 M HCl solution for 14 days. XRD and EIS data were collected before and after the test.

Electrochemical Fe^{2+}/Fe^{3+} Redox Tests

Cyclic voltammetry (CV) tests were carried out using a three-electrode setup with a glassy carbon working electrode, an Ag|AgCl (sat. KCl) reference electrode, and a graphite felt strip counter electrode. A CHI 1140D electrochemical workstation (CH Instrument, Austin, TX) was used to collect the CV curves. An aqueous solution containing 0.05 M $FeCl_2$ and 0.05 M $FeCl_3$ was used as electrolyte. A potential scan rate of $50mV\cdot s^{-1}$ was used to obtain the CV scans.

Crossover and Contamination of Fe^{2+}/Fe^{3+} Ectrolyte Tests

A 10-hour experiment was conducted to verify potential crossover between the electrolyte and product chambers. An aqueous solution containing 0.05 M $FeCl_2$ and 0.05 M $FeCl_3$ was used as the electrolyte. The feed consisted of an aqueous solution containing 0.05M of NaCl, LiCl, KCl, $MgCl_2$, and $CaCl_2$, respectively, with 0.01M HCl as the product solution. Samples were collected and analyzed using ICP-OES after 10 hours of operation at an applied voltage of 5 V.

DFT Calculation

Density functional theory (DFT) simulations were performed with the Quantum ESPRESSO package[2]. The Generalized Gradient Approximation (GGA) approach with the Perdew-Burke-Ernzerhof (PBE) functional and Projector-Augmented Wave (PAW) sets from PS library 0.3.1 were adopted for structure optimization and energy calculation[3]. The LSTH structure was constructed based on the previous experimental results[1]. The slab model consists of a four-layer close-packed (001) surface with a vacuum layer of 20 Å, where the top layer was SrO_2 terminated[4]. The bulk Li diffusion was proposed to diffuse to the nearest Li site in the unit cell with Ta center. The corresponding transition state searches were conducted with the Nudged Elastic Band (NEB) method. A plane-wave function cutoff of 55 Ry with a charge density cutoff of 550 Ry was used to initiate the calculations. The Monk horst-pack k-point meshes were set as 6 × 6 × 1 (for slab) and 6 × 6 × 6 (for bulk cell) with a thermal smearing of 0.01 Ry. All the energies were obtained after the structure relaxation.

Lithium Extraction Tests

A four-chamber rm-ED cell was assembled, consisting of a lab-made LSTH membrane, two commercial anion exchange membranes (AEMs, Fumasep FAS-PET-130), two graphite endplates, and rubber gaskets. The active geometric area of the LSTH membrane was 0.5 cm^2. Cell voltage was applied and recorded using a CHI 1140C electrochemical workstation. The conductivities of the product and feed streams were measured using a Mettler Toledo 230 conductivity

meter and an Inlab 741-ISM conductivity probe. Initially, a 10 ml solution containing 0.01 M HCl was used as the product stream for providing necessary ion conductivity while avoiding interference in the quantification of extracted lithium and other competing cations. The electrolyte contained 0.05 M $FeCl_3$ and 0.05 M $FeCl_2$ unless otherwise specified. All liquid flow rates were maintained at 30 ml·min^{-1}.

The average Li^+ extraction rate (LER, in mmol·h^{-1}·m^2) was calculated using Eq. 1:

$$\mathrm{LER} = \frac{(c_{Li^+} - c_{Li^+,0}) \times V_{product}}{A_c \times t} \quad (1)$$

where c_{Li^+} and $c_{Li^+,0}$ are the final and initial Li^+ concentrations in the product solution, respectively, as determined by ICP-OES (THERMO FISHER, USA) analyses. $V_{Product}$ represents the total volume of the product solution (20 mL in this work). A_c denotes the LSTH membrane area, and t is the experiment duration.

The energy consumption per mole of lithium extraction (EC, in kWh·mol^{-1}) was calculated using Eq. 2:

$$\mathrm{EC} = \frac{V \times C}{(c_{Li^+} - c_{Li^+,0}) \times V_{product}} \quad (2)$$

where V represents the applied voltage, and C is the total charge transfer during the experiment, as recorded by the electrochemical workstation.

The Li^+ purity in the product was calculated using Eq. 3:

$$Li^+ \text{ purity} = \frac{c_{Li^+}}{c_{Li^+} + c_{M^+}} \quad (3)$$

where c_{Li^+} and c_{M^+} are the concentrations of Li^+ and all other competing cations in the product solution, respectively, as determined by ICP-OES analyses.

The lithium enrichment factor was calculated using Eq. 4:

$$\text{Enrichment factor} = \frac{c_{Li^+}}{c_{Li^+,feed}} \quad (4)$$

where c_{Li^+} and $c_{Li^+,feed}$ represent the concentration of Li^+ in the product and feed solutions, respectively, as determined by ICP-OES analyses.

The charge efficiency (CE) was calculated using Eq. 5:

$$\mathrm{CE} = \frac{(c_{Li^+} - c_{Li^+,0}) \times V_{product} \times F}{C} \quad (5)$$

where F represents the Faraday constant.

Solar-Powered Lithium Extraction Tests

A commercial solar panel (Eujgoov, Polysilicon Solar Cell, 6.0 V, 1 W) and the rm-ED cell were integrated for demonstrating sustainable and eco-friendly lithium extraction. A solar simulator (PerfectLight PLS-SXE300C) with an intensity of 137 mW·cm^{-2} was employed for illuminating the solar panel to power the lithium extraction process. The rm-ED cell employed a 0.05 M $FeCl_3$ and 0.05 M $FeCl_2$ mixed solution as electrolyte and simulated seawater as brine feed stream, with all liquid flow rates maintained at 30 ml·min^{-1} during operation.

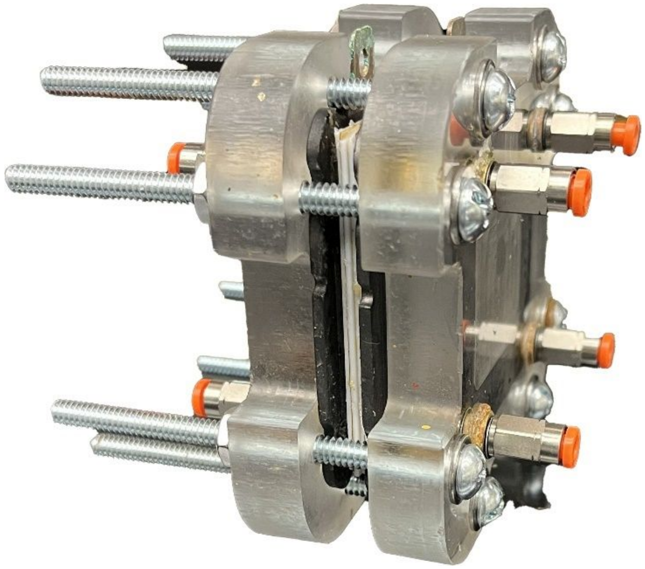

Figure S1. Photography of an assembled rm-ED cell for lithium extraction.

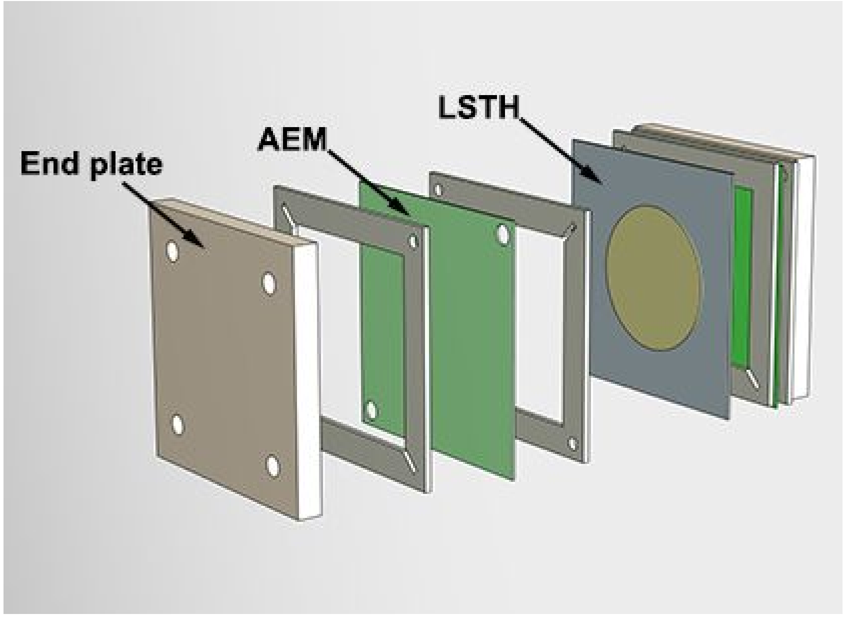

Figure S2. Components of a rm-ED cell structure.

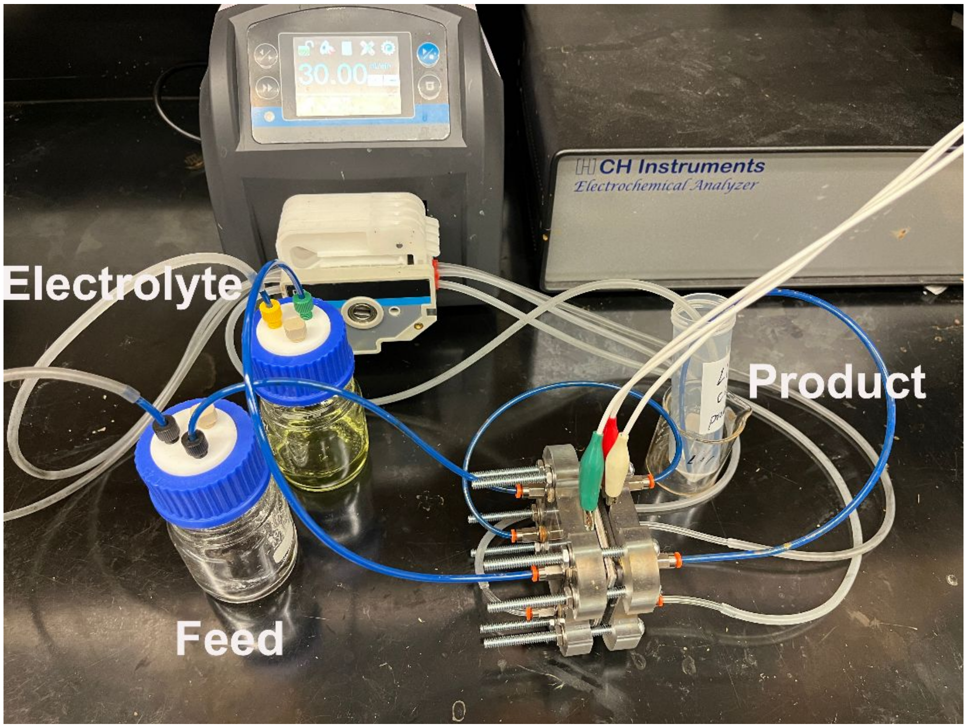

Figure S3. Photography of a rm-ED cell and lithium extraction system setup for experiments.

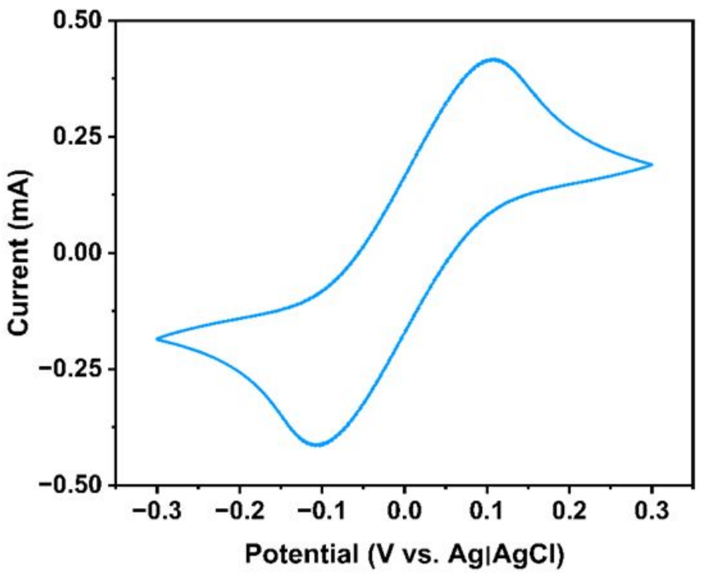

Figure S4. Cyclic voltammetry for Fe^{2+}/Fe^{3+} electrochemical redox process, obtained in electrolyte containing 50 mM $FeCl_3$ and 50 mM $FeCl_2$ with a scan rate of 50 $mV \cdot s^{-1}$.

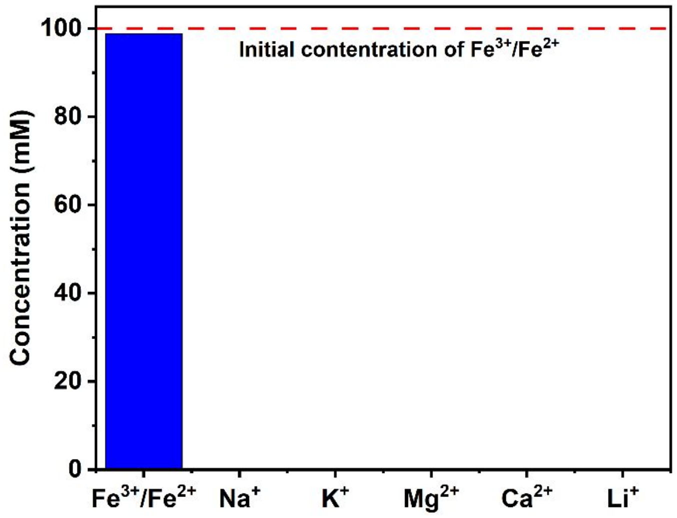

Figure S5. Elemental composition of the electrolyte after 10 hour of operation.

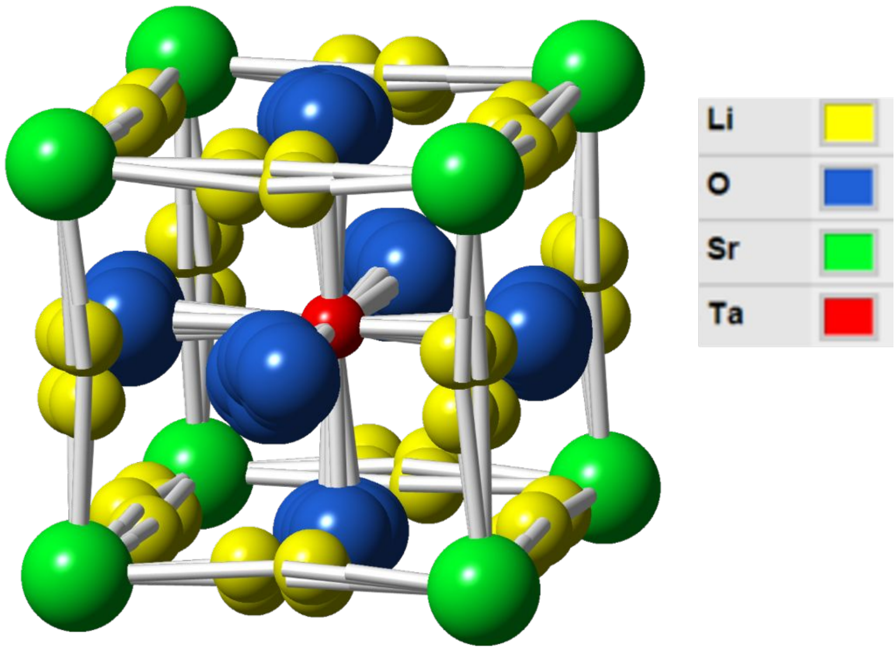

Figure S6. Pervoskite lattice structure of LSTH, as determined by XRD characterization.

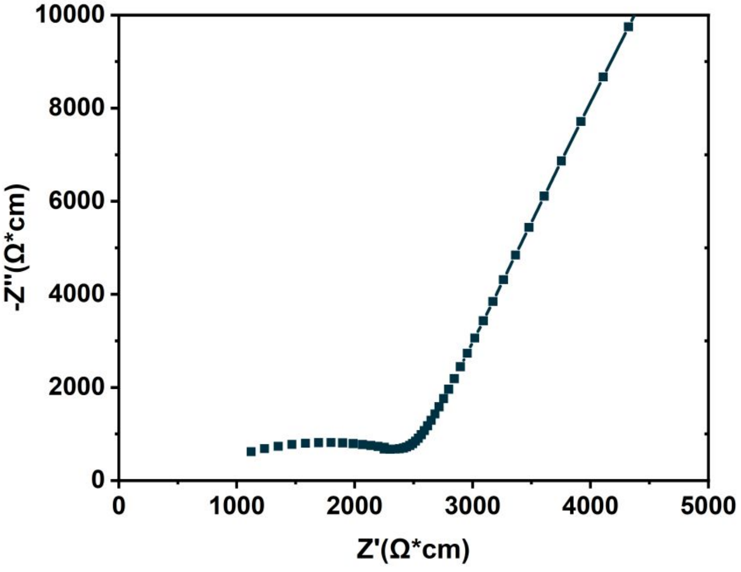

Figure S7. EIS data of LSTH membrane.

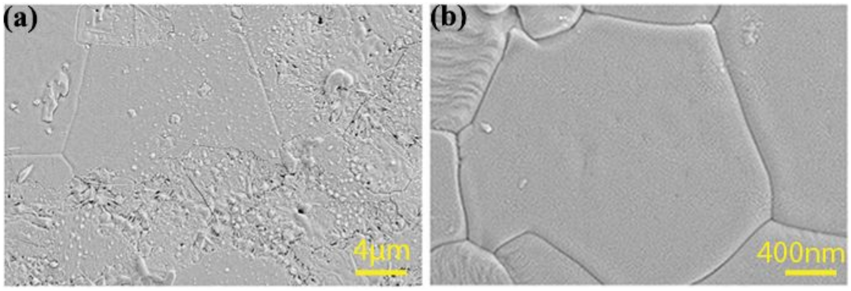

Figure S8. (a-b) SEM images of the surface of LSTH membrane at different magnifications

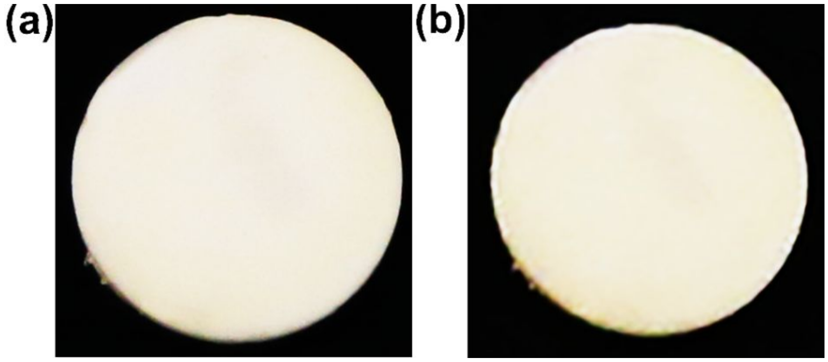

Figure S9. Photograph of LSTH membrane (a) before and (b) after 14 days of chemical stability test in 0.1 M HCl solution.

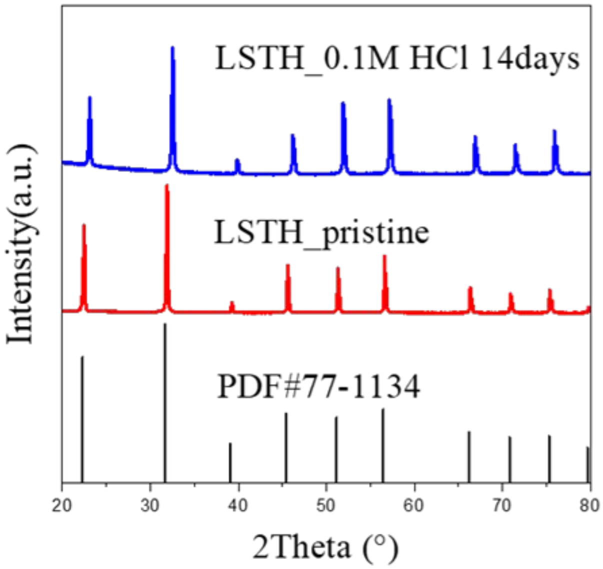

Figure S10. XRD data of LSTH before and after extended chemcial stability test in acidic environment.

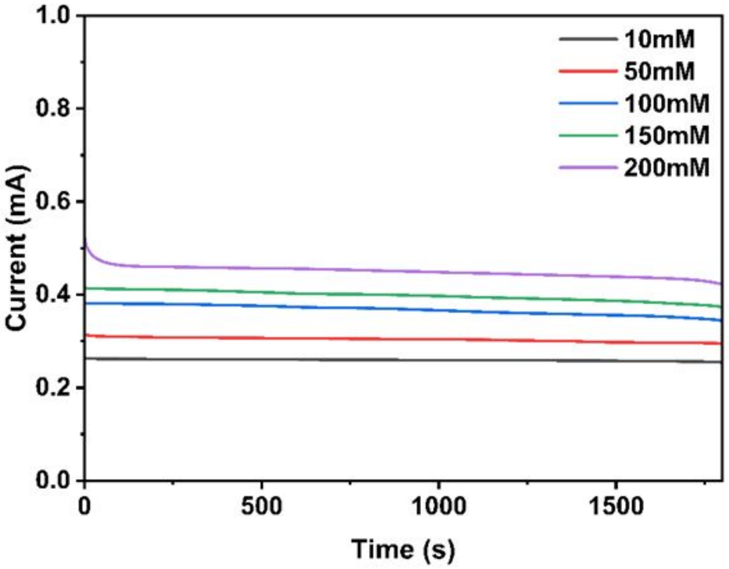

Figure S11. Measured rm-ED cell current as function of time, collected in lithium extraction experiments with different Li^{+} concentrations in the feed and an applied voltage of 5 V.

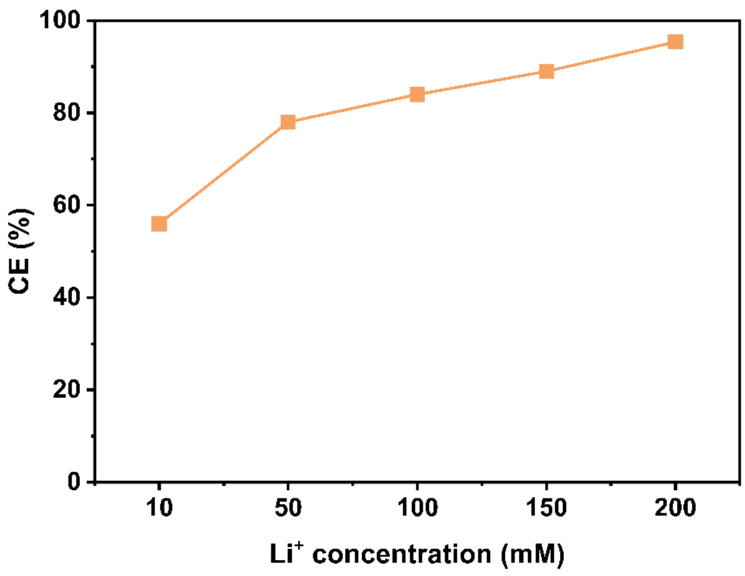

Figure S12. Determined charge efficiency in the lithium extraction experiments with different Li^{+} concentrations in the feed and an applied voltage of 5 V.

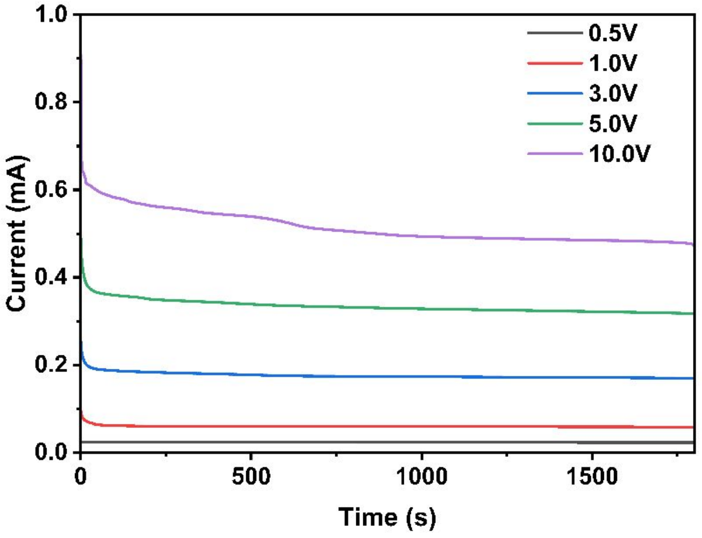

Figure S13. Measured rm-ED cell current as function of time, collected in lithium extraction experiments with 50 mM LiCl in the feed and different cell voltages.

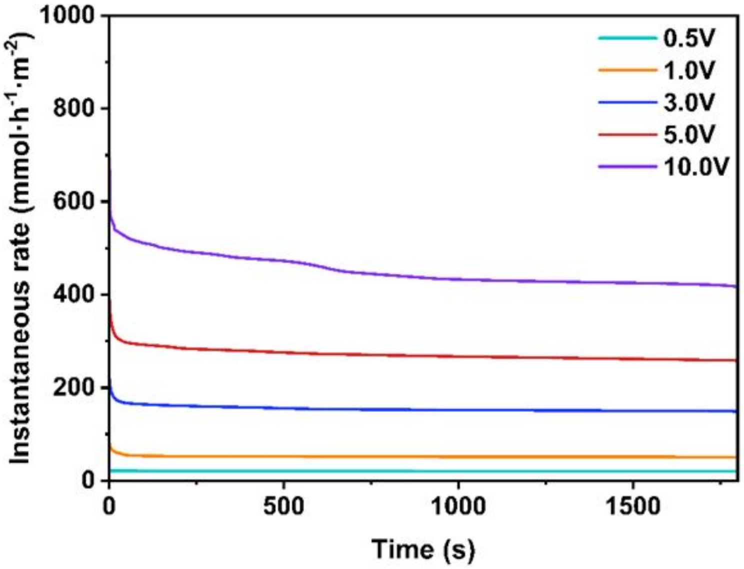

Figure S14. Instantaneous lithium extraction rate as function of time, calculated from rm-ED cell I-t data collected in lithium extraction experiments with 50 mM LiCl in the feed and different cell voltages.

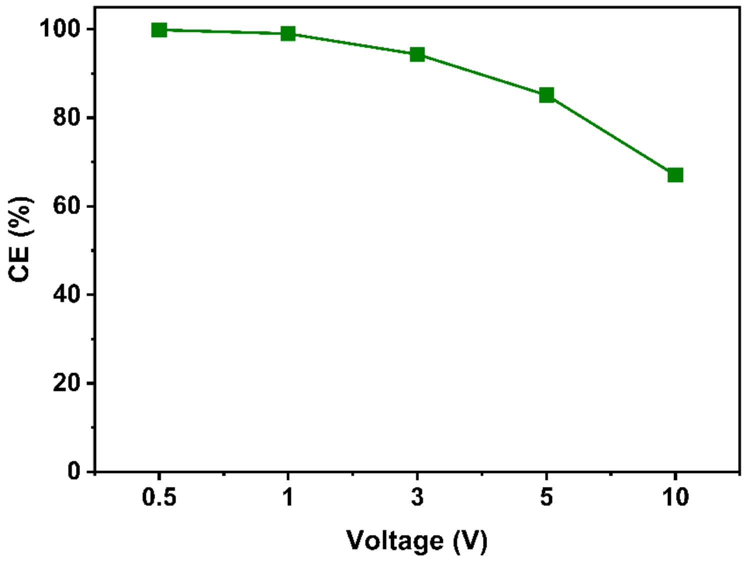

Figure S15. Determined charge efficiency in the lithium extraction experiments with 50mM LiCl and different cell voltages.

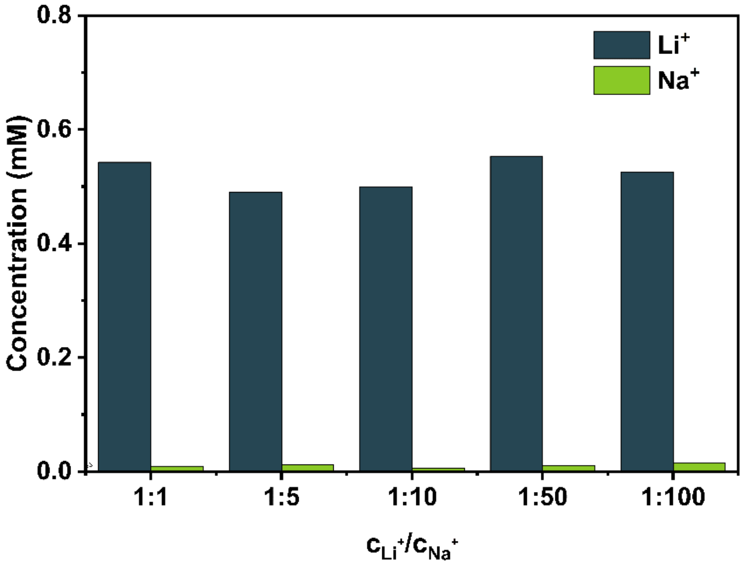

Figure S16. ICP-OES measured concentrations of Li^+ and Na^+ in the product of extraction experiments with different Li^+/Na^+ ratio in the feed (Li^+ maintained at 50 mM) and an applied voltage of 5V.

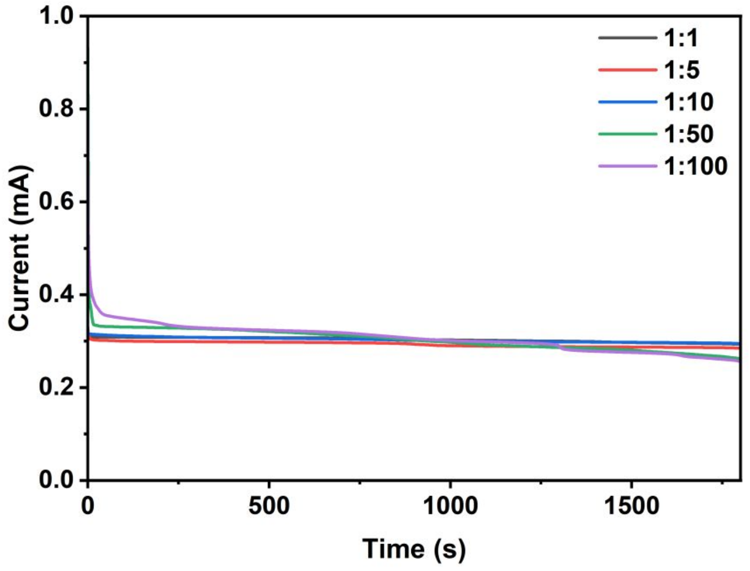

Figure S17. Measured rm-ED cell current as function of time, collected in lithium extraction experiments with different Li^+/Na^+ ratio in the feed (Li^+ maintained at 50 mM) and an applied voltage of 5V.

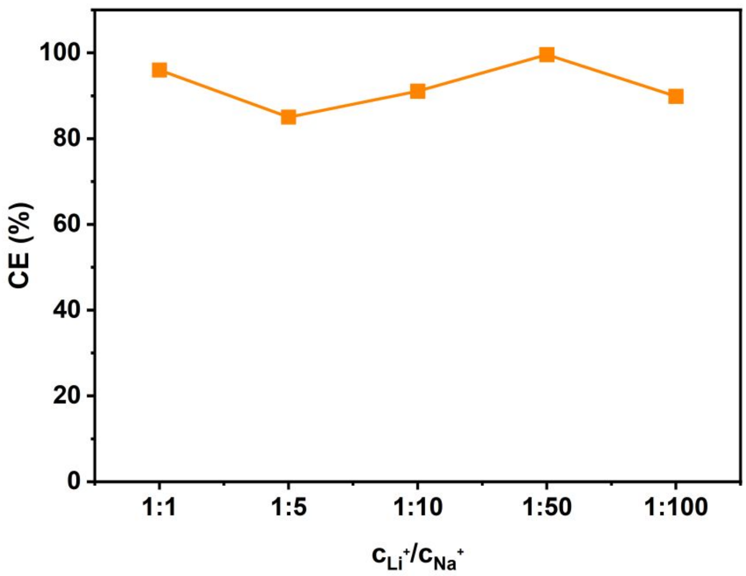

Figure S18. Determined charge efficiency in the lithium extraction experiments with different Li^+/Na^+ ratio in the feed (Li^+ maintained at 50 mM) and an applied voltage of 5V.

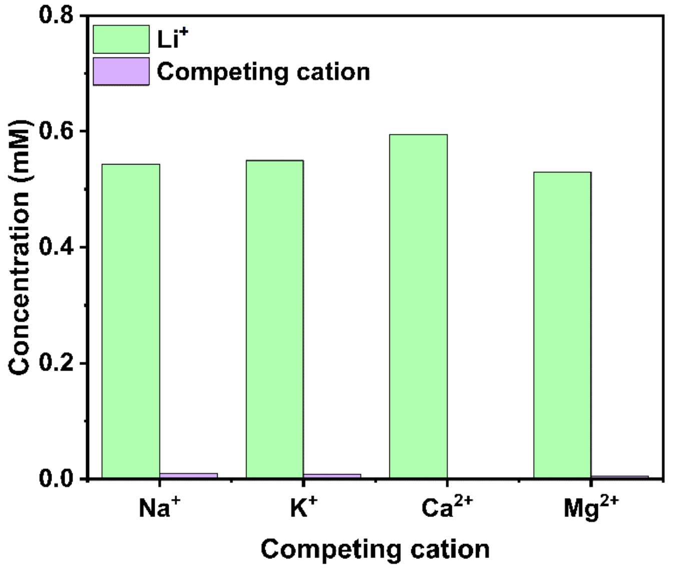

Figure S19. ICP-OES measured concentrations of Li^+ and competing cations in the extraction product, with various competing ions (Na^+, K^+, Ca^{2+}, Mg^{2+}) in the feed solution (all cation concentrations fixed at 50 mM) under an applied voltage of 5V.

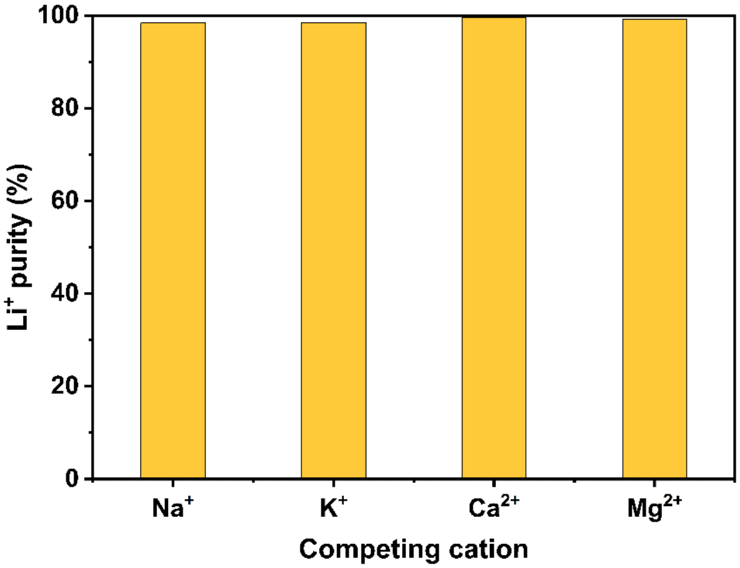

Figure S20. Li^+ purity in the extraction product from experiments with with various competing ions in the feed solution (all cation concentrations fixed at 50 mM) under an applied voltage of 5V.

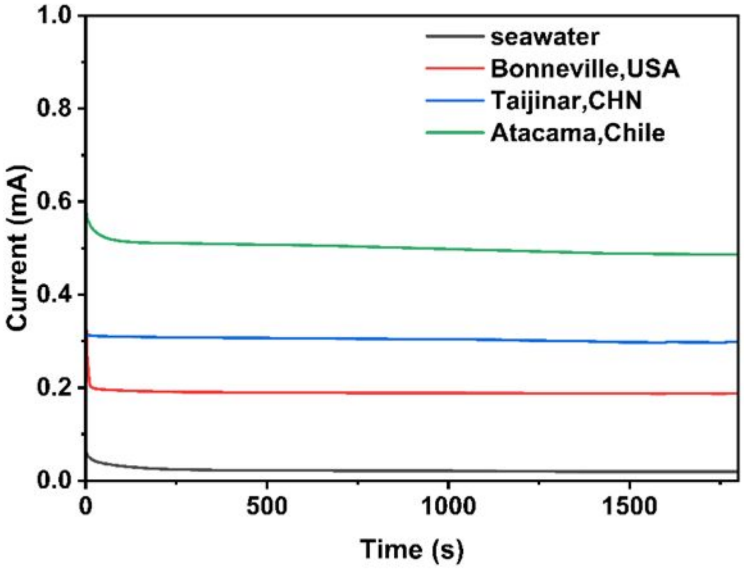

Figure S21. Measured rm-ED cell current as function of time, collected in lithium extraction experiments with different simulated water sources as the feed and an applied voltage of 5 V.

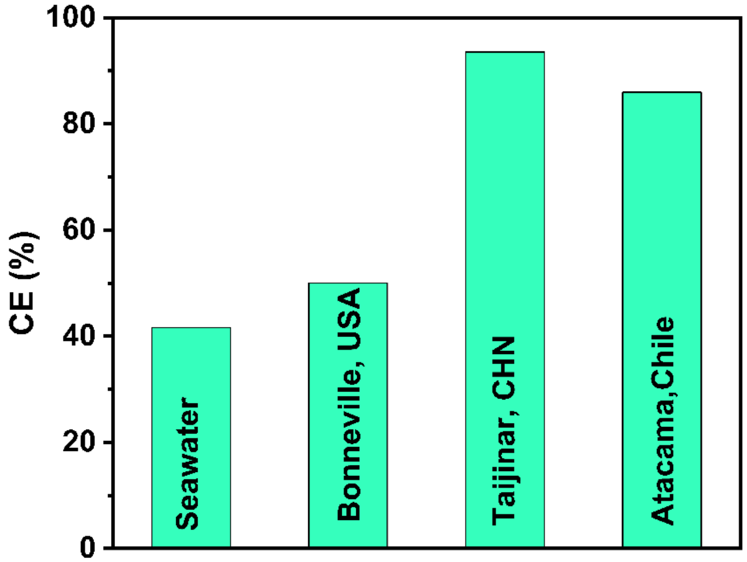

Figure S22. Determined charge efficiency in the lithium extraction experiments using different simulated water sources as the feed and an applied voltage of 5V.

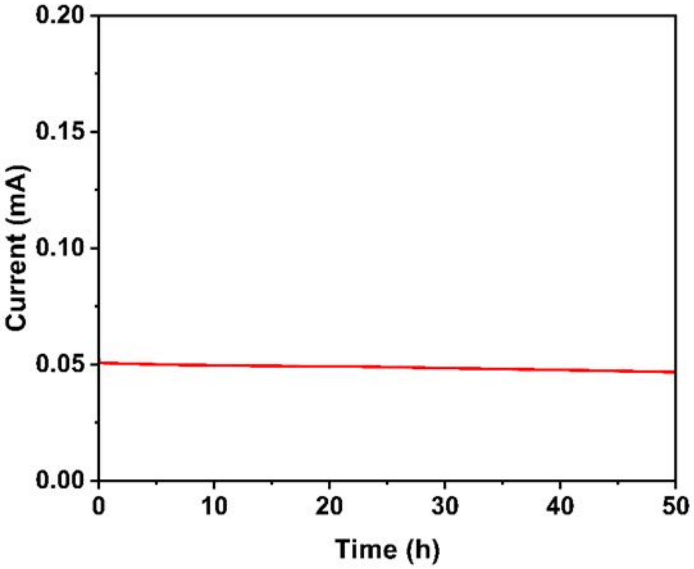

Figure S23. Measured rm-ED cell current as function of time for an extended period of 50 hrs, collected in lithium extraction experiment from seawater with an applied voltage of 5 V.

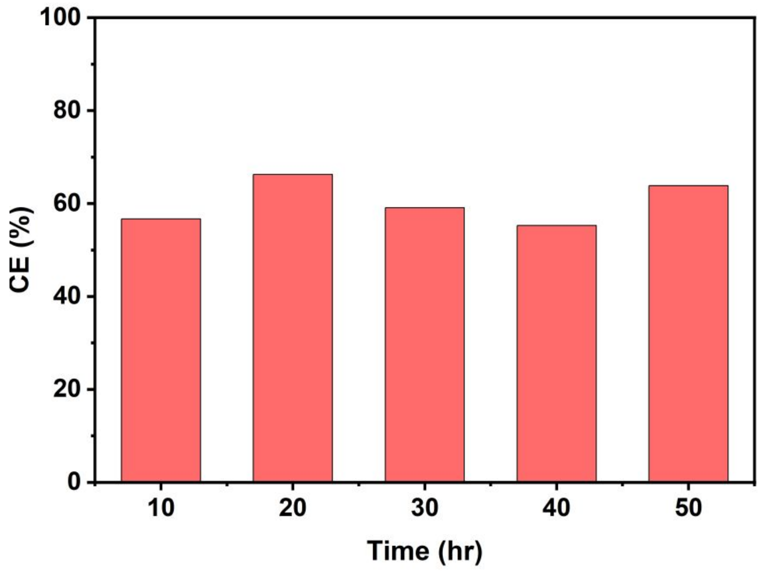

Figure S24. Determined charge efficiency as function of time in the lithium extraction experiments using seawater as the feed and an applied voltage of 5V.

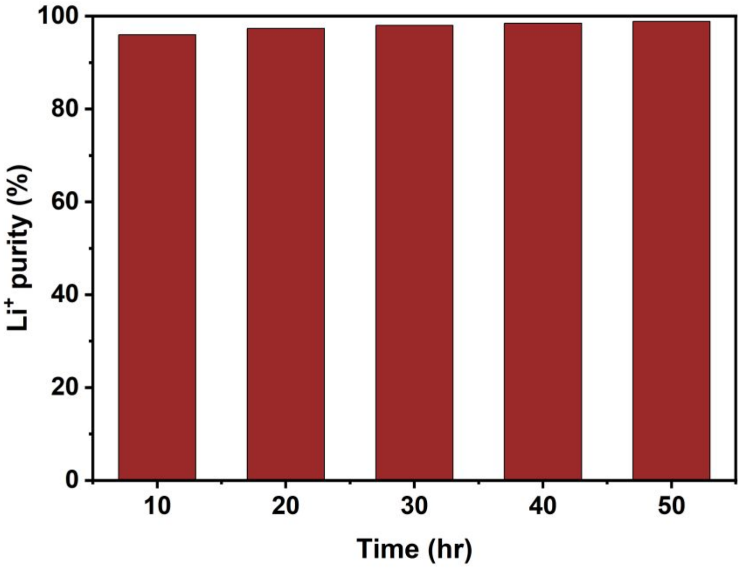

Figure S25. Li^+ purity in the product as function of time from seawater extraction powered by solar panel.

Table S1. Lattice sites and occupancies of each element in a unit LSTH cell.

	**x**	**y**	**z**	**Occ.**
Sr	0	0	0	0.411
Li	0	0.042	-0.412	0.016
Hf	0.5	0.5	0.5	0.25
Ta	0.5	0.5	0.5	0.75
O	0	0.462	0.51	0.121

Table S2. Experimental conditions and key performance results.

	Applied Voltage(V)	Average Current (mA)	Feed		Product(10ml)		Charge transfer (C)	Energy Consumption (kWh·mol^{-1})
			Li^{+}(mM)	Na^{+}(mM)	Li^{+}(mM)	Na^{+}(mM)		
10mM	5.0	0.23	10	/	0.24	/	0.41	0.23
50mM	5.0	0.33	50	/	0.51	/	0.60	0.17
100mM	5.0	0.39	100	/	0.63	/	0.71	0.16
150mM	5.0	0.41	150	/	0.69	/	0.74	0.15
200mM	5.0	0.45	200	/	0.80	/	0.81	0.14
0.5V	0.5	0.02	50	50	0.04	/	0.04	0.01
1.0V	1.0	0.07	50	50	0.13	0.001	0.13	0.03
3.0V	3.0	0.21	50	50	0.37	0.002	0.38	0.09
5.0V	5.0	0.34	50	50	0.51	0.002	0.60	0.17
10.0V	10.0	0.57	50	50	0.72	0.007	1.02	0.40
1:1	5.0	0.33	50	50	0.54	0.009	0.60	0.16
1:5	5.0	0.32	50	250	0.49	0.012	0.58	0.17
1:10	5.0	0.33	50	500	0.50	0.006	0.60	0.16
1:50	5.0	0.33	50	2500	0.55	0.010	0.60	0.15
1:100	5.0	0.34	50	5000	0.53	0.015	0.61	0.17

Table S3. Concentrations of major cations in different water sources.

Brine source	Na^+(mM)	K^+(mM)	Ca^{2+}(mM)	Mg^{2+}(mM)	Li^+(mM)
Taijinar, China[5]	2449	112.5	4.9	831.1	44.6
Atacama, Chile[6]	3958	603.6	11.2	397	226.2
Bonneville, USA[7]	3610	127.9	1.4	164.6	8.14
Sea water average[8, 9]	464.5	1.01	1.02	52.6	0.0243

REFERENCE

1. Sun, D.; Wu, N.; Qin, C.; White, R.; Huang, K. Synthesis and Characterization of Impurity-Free Li6/16Sr7/16Ta3/4Hf1/4O3 Perovskite as a Solid-State Lithium-Ion Conductor. *Energy Technology* **2023**, *11* (6), 2201455.
2. Giannozzi, P.; Baroni, S.; Bonini, N.; Calandra, M.; Car, R.; Cavazzoni, C.; Ceresoli, D.; Chiarotti, G. L.; Cococcioni, M.; Dabo,I.; et al. QUANTUM ESPRESSO: a modular and open-source software project for quantum simulations of materials. *J Phys Condens Matter* **2009**, *21* (39), 395502.
3. Dal Corso, A. Pseudopotentials periodic table: From H to Pu. *Computational Materials Science* **2014**, *95*, 337-350.
4. Yin, Y.; Wang, J.; Zhu, H.; Lv, K.; Wu, X. S. Structural distortion and charge redistribution in SrTiO3 (111) polar surfaces. *Vacuum* **2015**, *120*, 83-88.
5. Sun, S.-Y.; Cai, L.-J.; Nie, X.-Y.; Song, X.; Yu, J.-G. Separation of magnesium and lithium from brine using a Desal nanofiltration membrane. Journal of Water Process Engineering 2015, 7, 210-217.
6. Ogawa, Y.; Koibuchi, H.; Suto, K.; Inoue, C. Effects of the Chemical Compositions of Salars de Uyuni and Atacama Brines on Lithium Concentration during Evaporation. Resource Geology 2014, 64 (2), 91-101.
7. Yan, G.; Wang, M.; Hill, G. T.; Zou, S.; Liu, C. Defining the challenges of Li extraction with olivine host: The roles of competitor and spectator ions. Proc Natl Acad Sci U S A 2022, 119 (31), e2200751119.
8. Li, C.; Li, Z.; Wu, T.; Luo, Y.; Zhao, J.; Li, X.; Yang, W.; Chen, X. Metallogenic Characteristics and Formation Mechanism of Naomugeng Clay-Type Lithium Deposit in Central Inner Mongolia, China. Minerals 2021, 11 (3), 238.
9. Li, Z.; Li, C.; Liu, X.; Cao, L.; Li, P.; Wei, R.; Li, X.; Guo, D.; Huang, K.-W.; Lai, Z. Continuous electrical pumping membrane process for seawater lithium mining. Energy & Environmental Science 2021, 14 (5), 3152-3159.
