# Supplementary material for: Ecofriendly, Highly Selective Lithium Extraction by Redox-Mediated Electrodialysis
Source: ACS Cent Sci. 2024 Nov 9;10(11):2119–24. doi: 10.1021/acscentsci.4c01373 (PMC11613207; doi:10.1021/acscentsci.4c01373)
Supplement: Supplementary file 2 — oc4c01373_si_002.pdf [file oc4c01373_si_002.pdf]

oc-2024-013735.R1

Name: Peer Review Information for "Eco-friendly, Highly Selective Lithium Extraction by Redox-mediated Electrodialysis"

First Round of Reviewer Comments

Reviewer: 1

Comments to the Author

The manuscript explores the use of Fe^{2+}/Fe^{3+} as auxiliary electrodes to prevent hydrogen and chlorine evolution reactions at the electrodes, thereby achieving a lower electrodialysis potential. The study demonstrates both feasibility and innovation. However, the characterization is incomplete, and the data processing lacks refinement. The manuscript requires the following revisions before it can be recommended for publication:

1. The authors refer to their redox-mediated electrodialysis process as an improved ED process, abbreviated as "RED," which is inappropriate. "RED" typically stands for reverse electrodialysis. It is recommended that the process be renamed rm-ED or Rm-ED to avoid confusion with the established RED process and prevent potential misunderstandings, especially in membrane society.

2. The selectivity of the anion exchange membrane (AEM) for cations and anions is limited. In the electrodialysis (ED) process, it is common for electrode solutions to permeate the ion exchange membrane (IEM) into the ED chamber, or for ions from the ED chamber to permeate the IEM and contaminate the electrode solution (Desalination 2010, 264, 268–288; AIChE Journal, 2008, 54, 3147–3159; Energy Environ. Sci., 2021, 14, 3152–3159). When using low-grade lithium resources with low lithium content and high impurity levels (e.g., seawater), the rates of electrode solution loss and contamination per unit of extracted lithium are significant. Additionally, in this study, the single cell voltage can reach up to 10V, which could exacerbate loss and contamination under such high electric fields. The authors should quantify the amount of iron ion leakage through the AEM into the lithium product and the extent of impurity ion permeation through the AEM into the electrode chamber. They should also propose viable solutions to address the loss and contamination of the electrode solution.

3. In the Rm-ED system described, the energy consumption ranges from 0.07 to 0.24 $kWh \cdot mmol^{-1}$, equivalent to 10,000 to 34,385 $kWh \cdot kg^{-1}$. This is three orders of magnitude higher than values reported in the literature. With such high energy consumption, the authors need to comment the feasibility of practical application of their method.

4. The selectivities for Li/Na, Li/Mg, Li/K, and Li/Ca should be evaluated and presented in a comprehensive graphical format.

5. The formula for Li^+ purity in equation 3 of the supplementary information is problematic, as it only considers sodium as an impurity. Consequently, Figure 3c, based on this formula, is unreliable, and the claim of achieving lithium purity as high as 99% is questionable. In real brine, alongside sodium, there are significant amounts of magnesium, potassium, and calcium. The authors should use inductively coupled plasma (ICP) analysis to quantify all impurities in the water samples, particularly Na, K, Mg, Ca, and Fe. Furthermore, they should convert the solution into a solid salt form (e.g., LiCl, Li_2CO_3, or others) and confirm the purity of the obtained salts.

6. The authors should perform cyclic voltammetry (CV) measurements on the ceramic membrane to confirm its electrochemical stability window and test its stability under high voltage conditions (e.g., over one week or longer).

7. The authors should measure the polarization curves of the Fe^{2+}/Fe^{3+} electrodes to determine the overpotential of the electrodes under different operating loads.

8. The studied system is an aquous system, and a voltage of 5 - 10 V is very high. I wonder how this is possible. The authors should measure the voltage distribution across the cell to determine how much of the applied 5-10V is consumed by the electrodes, the AEM, and the ceramic membrane, respectively.

Reviewer: 2

Comments to the Author

This work designs a redox-mediated electrodialysis method to extract lithium from brines. They used a dense ceramic Li6/16Sr7/16Ta3/4Hf1/4O3 (LSTH) perovskite membrane, demonstrating high selectivity for lithium ions. Despite these advancements, several issues require further clarification:

(1). There appears to be a discrepancy in the lithium extraction rate and energy consumption data. Specifically, the manuscript states on page 3 (lines 28-31) that "…lithium extraction rate of 172 mmol·h-1·m-2 and an energy consumption of 0.102 kWh·mmol-1 were achieved when a 10 mM LiCl brine stream was fed to the RED cell"). Upon calculation, these figures seem contradictory given the supporting information provided in the "Lithium Extraction Tests" section (Figure S10), as detailed below:

As shown in the "Lithium Extraction Tests" section in supporting information file: the area of LSTH membrane was 0.5 cm2, the volume of product solution was 20 mL, the operation time was 0.5h (Figure S10) and the Li+ extraction rate (LER) and energy consumption (EC) were calculated by Eq. 1 and 2.

With the description shown in Page 3 in the manuscript and the data shown in Figure 2a and b, we can get the following calculations (The data provided by the authors are bolded):

(cLi+—cLi+,0) × Vproduct = LER × Ac × t =172 mmol·h-1·m-2 × 0.5 × 10-4 m2 × 0.5 h = 0.0043 mmol (By using Eq. 1)

V × C = EC × (cLi+—cLi+,0) × Vproduct = 0.102 kWh·mmol-1 × 0.0043 mmol = 0.0004386 kWh = 0.4386 Wh (By using Eq. 2)

The cell voltage was 5 V (Shown in lines 23-24, Page 3 in manuscript), so the value of the total charge transfer "C" is 0.08772 Ah. During the operation time 0.5 h, the average current should be 0.17544 A (Equal to 175.44 mA).

However, as shown in Figure S10, when the lithium concentration in the feed was 10 mM, the current was more than 0.2 mA but lower than 0.3 mA. These discrepancies require further explanation or correction.

(2). I recommend including tables in the supporting information file to detail the data used in Figure 2. These tables should list the concentrations of Li+ and Na+ in both the feed and recovery solutions before and after lithium extraction, applied voltage, and total charge transfer during the operation, to enhance data transparency and reproducibility.

(3). There is an inconsistency in the current profiles under identical conditions of lithium concentration and voltage in Figures S10 and S12. Figure S10 shows a smooth current curve (red line), whereas Figure S12 depicts an initial decrease followed by a stable current (green line). The reasons for these differences should be investigated and clarified.

(4). The manuscript suggests that increased energy consumption is due to higher cell currents leading to greater ohmic losses and reduced charge efficiency, as seen in Figure S14. However, it is unclear why the charge efficiency (CE) at 0.5 V is lower than at 1 V. A more detailed analysis would be beneficial here to understand the impact of voltage on CE.

(5). There seems to be an error in Figure S18, where the operation times for seawater and Bonneville are shorter than those for Taijiner and Atacama. Please verify these data points and correct any potential inaccuracies.

(6). The ion concentration data from different brines cited in Table S2 rely solely on Reference 21. This citation might not provide sufficient validation for the data presented. A thorough review of the literature or additional experimental verification would strengthen the credibility of these values. And is “Aracama” spelled wrong? Is it “Atacama”?

Reviewer: 3

Comments to the Author

Authors reported a redox-mediated (Fe3+/Fe2+) electrodialysis method to selectively extract Li from brines. By using a dense ceramic membrane, the authors showed a continuous operation system with good extraction rates, low energy consumption, and good Li selectivity. Thus, this work is believed to draw the interest of broad researchers and is suitable to be published in ACS Central Science. Before publishing, a few small aspects can be improved.

1. Utilizing electrodialysis with ceramic membranes to realize lithium extraction is not new. How good is this work compared to other works using electrodialysis (e.g. 1. Lithium Metal Extraction from Seawater. 2. A low-cost anodic catalyst of transition metal oxides for lithium extraction from seawater) and other methods, such as electrochemical intercalation, adsorption, solvent extraction, and ion exchange?

2. People have reported electrodialysis method coupled with OER for lithium extraction (A low-cost anodic catalyst of transition metal oxides for lithium extraction from seawater). Could the authors provide some insightful comments on choosing redox pairs to complete the lithium extraction loop, in terms of efficiency, energy assumption, and environmental impact, cost, and scalability?

3. Figure 3d. the authors attributed the decrease in the extraction rate to the decrease of the lithium concentrations in the feed brine stream. But this could give a static dropping of the extraction rate. However, from Figure 3d, the extraction retention suddenly dropped in 20 – 30 h range. A better explanation should be provided.

Author's Response to Peer Review Comments:

Dear Editor Prof. Zheng,

Thank you for giving us the opportunity to revise our manuscript “Eco-friendly, Highly Selective Lithium Extraction by Redox-mediated Electrodialysis” in ACS Central Science. We sincerely thank the time and effort that you and all reviewers dedicated to providing valuable comments on our manuscript.

We have addressed all the reviewers’ valuable questions and comments and have incorporated the suggestions made by the reviewers in this revised manuscript. The

changes are tracked by being highlighted in the revised manuscript. Enclosed below please find our point-by-point response to the reviewers' comments. We hope this revision makes our paper acceptable to your prestigious journal.

Sincerely yours,

Zhenmeng Peng

Point-by-Point Response to Reviewers' Comments

Reviewer: 1

Recommendation: Reconsider after major revisions noted.

Comments:

The manuscript explores the use of Fe^{2+}/Fe^{3+} as auxiliary electrodes to prevent hydrogen and chlorine evolution reactions at the electrodes, thereby achieving a lower electrodialysis potential. The study demonstrates both feasibility and innovation. However, the characterization is incomplete, and the data processing lacks refinement. The manuscript requires the following revisions before it can be recommended for publication:

1. The authors refer to their redox-mediated electrodialysis process as an improved ED process, abbreviated as "RED," which is inappropriate. "RED" typically stands for reverse electrodialysis. It is recommended that the process be renamed rm-ED or RmED to avoid confusion with the established RED process and prevent potential misunderstandings, especially in membrane society.

Response: We sincerely thank the reviewer for the valuable suggestion and acknowledge that the use of improper abbreviation could indeed lead to confusion. To address this concern, we have revised the manuscript by replacing all 'RED' with 'rm-ED' in order to enhance clarity and readability for readers. For example, in the introduction, "Herein, we report a redox-mediated electrodialysis (rm-ED) method designed for energy-efficient, continuous, direct extraction of lithium, with purity exceeding 99%, from various brine sources. This method employs a dense ceramic $Li_{6/16}Sr_{7/16}Ta_{3/4}Hf_{1/4}O_3$ (LSTH) perovskite membrane that exclusively permits lithiumion exchange at the interface and facilitates transport through its lattice structure, achieving exceptional lithium selectivity. Moreover, it does not consume any chemicals, generates no waste, and can be powered by renewable energy, ensuring eco-friendliness."

2. The selectivity of the anion exchange membrane (AEM) for cations and anions is limited. In the electrodialysis (ED) process, it is common for electrode solutions to permeate the ion exchange membrane (IEM) into the ED chamber, or for ions from the ED chamber to permeate the IEM and contaminate the electrode solution (Desalination 2010, 264, 268–288; AIChE Journal, 2008, 54, 3147–3159; Energy Environ. Sci., 2021, 14, 3152–3159). When using low-grade lithium resources with low lithium content and high impurity levels (e.g., seawater), the rates of electrode solution loss and contamination per unit of extracted lithium are significant. Additionally, in this study, the

single cell voltage can reach up to 10V, which could exacerbate loss and contamination under such high electric fields. The authors should quantify the amount of iron ion leakage through the AEM into the lithium product and the extent of impurity ion permeation through the AEM into the electrode chamber. They should also propose viable solutions to address the loss and contamination of the electrode solution.

Response: We sincerely appreciate the reviewer's insightful comment and suggestion. We agree that for practical applications, the potential crossover of undesired ions through the IEMs between the ED chamber and the electrode solution is a critical concern. To evaluate this, we conducted a 10-hour experiment to monitor ion permeation in our system.

At the start of our experiment, the electrolyte contained 100 mM Fe^{3+}/Fe^{2+} with no other metal cations present. After 10 hours of operation, the concentration of Fe^{3+}/Fe^{2+} was measured to be 98.9 mM, with only trace amounts of other metal cations detected (Figure S5). Additionally, the product stream initially contained only HCl solute, and throughout the 10-hour test, the Fe^{3+}/Fe^{2+} concentration in the product stream remained minimal, with no detectable increase over time (Figure R1). This indicates that the undesired ion crossover remained negligible during the operation.

We believe the minor variations in the measured ions' concentrations observed before and after the operation are primarily due to measurement errors, as the concentrations were near the detection limits of our ICP-OES system. Additionally, we measured the voltage distribution across the rm-ED device and found that only 0.06 V was applied across AEM when the total voltage was set to 5 V (see response to comment #8 for measurement details). At such a low voltage, the likelihood of cation crossover between the electrolyte and product chambers through the AEM is minimal. These results demonstrate a good performance of the AEMs (Fumasep FAS-PET-130) under the operating conditions in this work, with minimal risk of Fe^{3+}/Fe^{2+} electrolyte crossover or contamination.

We have added Figure S5 and a section in the revised Supporting Information to provide further details:

"Crossover and Contamination of Fe^{2+}/Fe^{3+} Electrolyte Tests

A 10-hour experiment was conducted to verify potential crossover between the electrolyte and product chambers. An aqueous solution containing 0.05 M $FeCl_2$ and 0.05 M $FeCl_3$ was used as the electrolyte. The feed consisted of an aqueous solution containing 0.05 M of NaCl, LiCl, KCl, $MgCl_2$, and $CaCl_2$, respectively, with 0.01 M HCl as the product solution. Samples were collected and analyzed using ICP-OES after 10 hours of operation at an applied voltage of 5 V."

To further suppress the potential crossover issue, one viable approach is to develop AEM with outstanding resistance to cations that can effectively inhibit their permeation to contaminate the neighbor chambers. Another viable approach is to switch to

ironchelate compound redox electrolyte. Due to their larger molecular size and lower diffusivity, iron-chelate compounds can be significantly less likely to pass through AEMs, thereby providing an additional safeguard against crossover and contamination.[1]

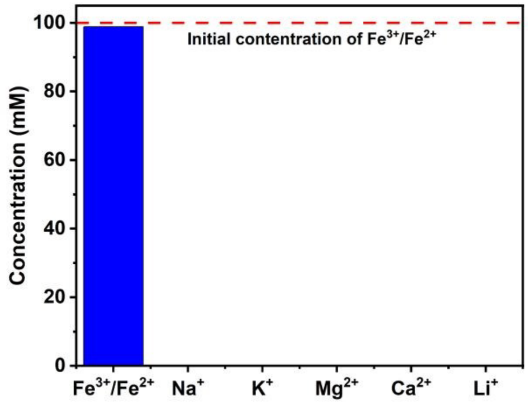

Figure S5. Elemental composition of the electrolyte after 10 hours of operation.

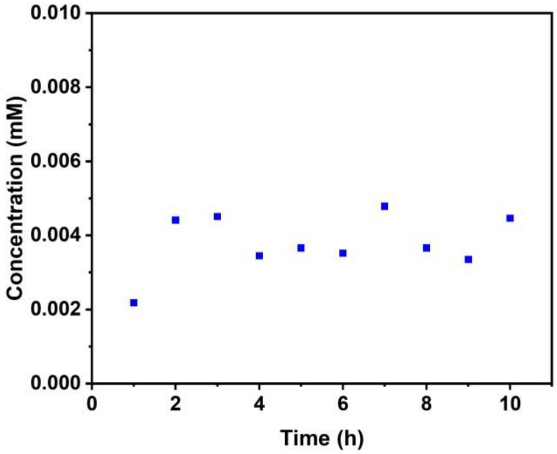

Figure R1. Concentration of Fe^{3+}/ Fe^{2+} in the product stream during 10-hour operation.

3. In the Rm-ED system described, the energy consumption ranges from 0.07 to 0.24 kWh·mmol⁻¹, equivalent to 10,000 to 34,385 kWh·kg⁻¹. This is three orders of magnitude higher than values reported in the literature. With such high energy consumption, the authors need to comment the feasibility of practical application of their method.

Response: We sincerely appreciate the reviewer's valuable question and apologize for the two errors introduced in our calculations. First, we overcalculated the energy consumption by three orders of magnitude due to a unit conversion mistake, calculating in kWh·mmol⁻¹ instead of the correct unit, kWh·mol⁻¹. Additionally, we initially used a 20 mL volume for the product stream, but later reduced it to 10 mL in most experiments to increase the lithium concentration and obtain more precise ICP-OES measurements. Unfortunately, we failed to update this volume change in our calculations, leading to

energy consumption values that were incorrectly reported as half of their actual values. These two errors together resulted in an overestimation of the energy consumption by approximately 500 times. After carefully reviewing the raw data and recalculating several times, we have corrected these errors in the revised manuscript. The lowest energy consumption in this work is now reported as 0.014 $kWh \cdot mol^{-1}$ obtained at an applied voltage of 0.5 V (Figure 2d), corresponding to 2 $kWh \cdot kg^{-1}$ of Li^{+}. This value is significantly lower than those reported for conventional electrodialysis methods.[2-4]

The revised data, along with the updated Figure 2, have been incorporated into the manuscript. Additionally, Table S2 has been added to the Supporting Information to provide a comprehensive summary of all experimental conditions and results. We have also attached an Excel spreadsheet detailing the raw data and calculations for full transparency. We believe these corrected values strongly demonstrate the feasibility of our method for practical applications, particularly due to its significant energy efficiency compared to existing technologies.

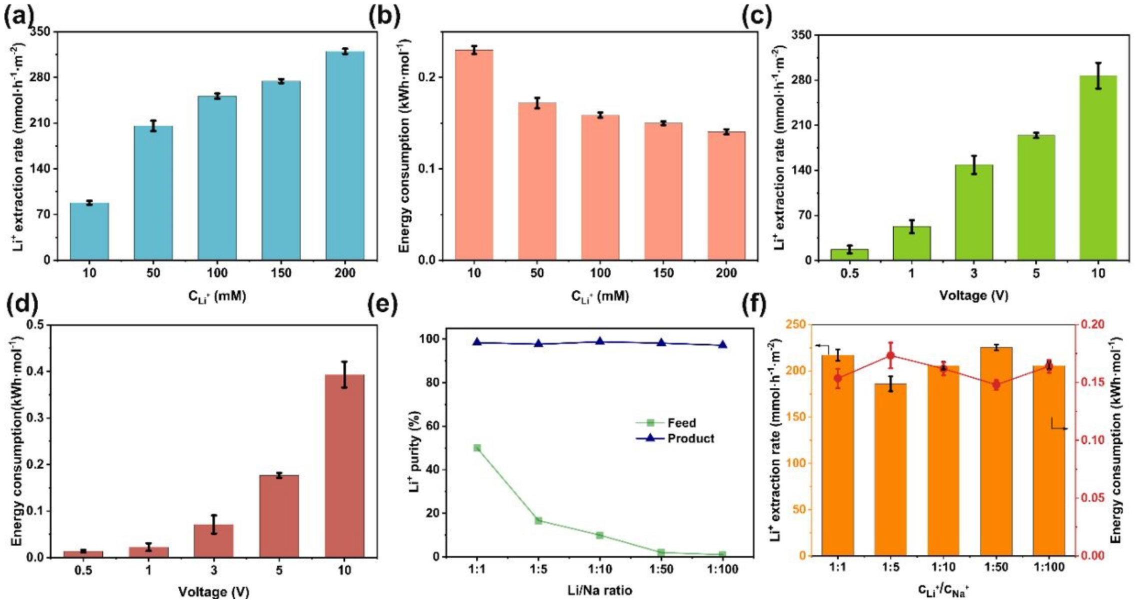

Figure 2. (a, b) Effects of Li-ion concentration in feed on average lithium extraction rate and energy consumption, with 5 V rm-ED cell voltage; (c, d) Effects of applied voltage on average rate and energy consumption for lithium extraction from 50 mM Liion feed; (e, f) Effects of Li^{+}/Na^{+} ratio in feed on lithium purity in product, average lithium extraction rate, and energy consumption, with Li-ion concentration in feed fixed at 50 mM and 5 V rm-ED cell voltage.

Table S2. Experimental conditions and key performance results.

	Feed	Product(10ml)	Energy

	Applied Voltage(V)	Average Current (mA)	Li^+(mM)	Na^+(mM)	Li^+(mM)	Na^+(mM)	Charge transfer (C)	Consumption ($kWh \cdot mol^{-1}$)
10mM	5.0	0.23	10	/	0.24	/	0.41	0.23
50mM	5.0	0.33	50	/	0.51	/	0.60	0.17
100mM	5.0	0.39	100	/	0.63	/	0.71	0.16
150mM	5.0	0.41	150	/	0.69	/	0.74	0.15
200mM	5.0	0.45	200	/	0.80	/	0.81	0.14
0.5V	0.5	0.02	50	/	0.04	/	0.04	0.01
1.0V	1.0	0.07	50	50	0.13	0.001	0.13	0.03
3.0V	3.0	0.21	50	50	0.37	0.002	0.38	0.09
5.0V	5.0	0.34	50	50	0.51	0.002	0.60	0.17
10.0V	10.0	0.57	50	50	0.72	0.007	1.02	0.40
1:1	5.0	0.33	50	50	0.54	0.009	0.60	0.16
1:5	5.0	0.32	50	250	0.49	0.012	0.58	0.17
1:10	5.0	0.33	50	500	0.50	0.006	0.60	0.16
1:50	5.0	0.33	50	2500	0.55	0.010	0.60	0.15
1:100	5.0	0.34	50	5000	0.53	0.015	0.61	0.17

4. The selectivities for Li/Na, Li/Mg, Li/K, and Li/Ca should be evaluated and presented in a comprehensive graphical format.

Response: We sincerely appreciate the reviewer's valuable suggestion and agree that presenting selectivity data for Li^+ against other competing cations will enhance the demonstration of the LSTH membrane's performance. In response, we have conducted additional experiments to evaluate the selectivity for Li/Mg, Li/K, and Li/Ca besides for Li/Na. The results from these tests, which used mixed solutions of 50 mM LiCl with 50 mM of either KCl, $CaCl_2$, or $MgCl_2$, have been added to the revised manuscript, as "To further assess the selectivity of the LSTH membrane against competing cations, we tested three mixed solutions containing 50 mM LiCl and 50 mM of either KCl, $CaCl_2$, or $MgCl_2$. The results demonstrated that the concentrations of the competing cations remained at extremely low levels in the products, indicating high selectivity for Li^+ (Figures S19 and S20)". As shown in the added Figures S19 and S20 in the revised SI, the LSTH membrane exhibited high selectivity for Li^+, with competing cations remaining at extremely low levels. A comprehensive graphical comparison of the selectivity for Li^+ against Na^+, Mg^{2+}, K^+, and Ca^{2+} has also been included for clarity.

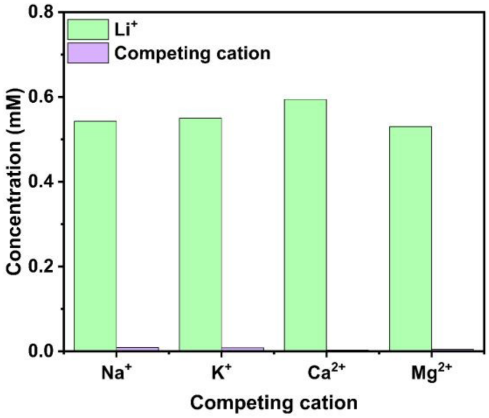

Figure S19. ICP-OES measured concentrations of Li^+ and competing cations in the extraction product, with various competing ions (Na^+, K^+, Ca^{2+}, Mg^{2+}) in the feed solution (all cation concentrations fixed at 50 mM) under an applied voltage of 5V.

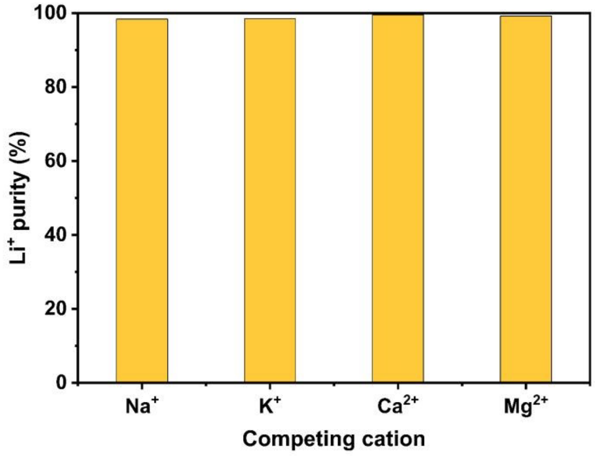

Figure S20. Li^+ purity in the extraction product from experiments with with various competing ions in the feed solution (all cation concentrations fixed at 50 mM) under an applied voltage of 5V.

5. The formula for Li^+ purity in equation 3 of the supplementary information is problematic, as it only considers sodium as an impurity. Consequently, Figure 3c, based on this formula, is unreliable, and the claim of achieving lithium purity as high as 99% is questionable. In real brine, alongside sodium, there are significant amounts of magnesium, potassium, and calcium. The authors should use inductively coupled plasma (ICP) analysis to quantify all impurities in the water samples, particularly Na, K, Mg, Ca, and Fe. Furthermore, they should convert the solution into a solid salt form (e.g., LiCl, Li_2CO_3, or others) and confirm the purity of the obtained salts.

Response: We sincerely appreciate the reviewer's valuable suggestion and agree that Equation 3 was confusing as originally presented. Our intention was to use purity to represent the molar percentage of Li^+ in the product stream relative to all other competing cations. The previous equation mistakenly used only c_{Na+}, as Na^+ was the sole competing cation in results shown in Figure 2.

In Figure 3, however, our purity calculations did already include Li^+ and all other competing cations in the experiments, including Na^+, K^+, Mg^{2+}, and Ca^{2+}. To clarify, we have revised Equation 3 in the Supporting Information as "The Li^+ purity in the product was calculated using Eq. 3:

$$\text{Li}^+\ \text{purity} = \frac{c_{Li+}}{c_{Li+} + c_{M+}} \quad (3)$$

where c_{Li+} and c_{M+} are the concentrations of Li^+ and all other competing cations in the product solution, respectively, as determined by ICP-OES analyses."

Additionally, we agree with the reviewer's suggestion that, considering the presence of various anions in real brines, it is more appropriate to convert the solution into a solid salt form and confirm the purity of the obtained salts. In response, we have converted all cations into their chloride forms (e.g., LiCl, NaCl) and have updated Figure 3 accordingly. This allows for a more accurate representation of the purity of lithium in the final product. The revised Figure 3 is included below.

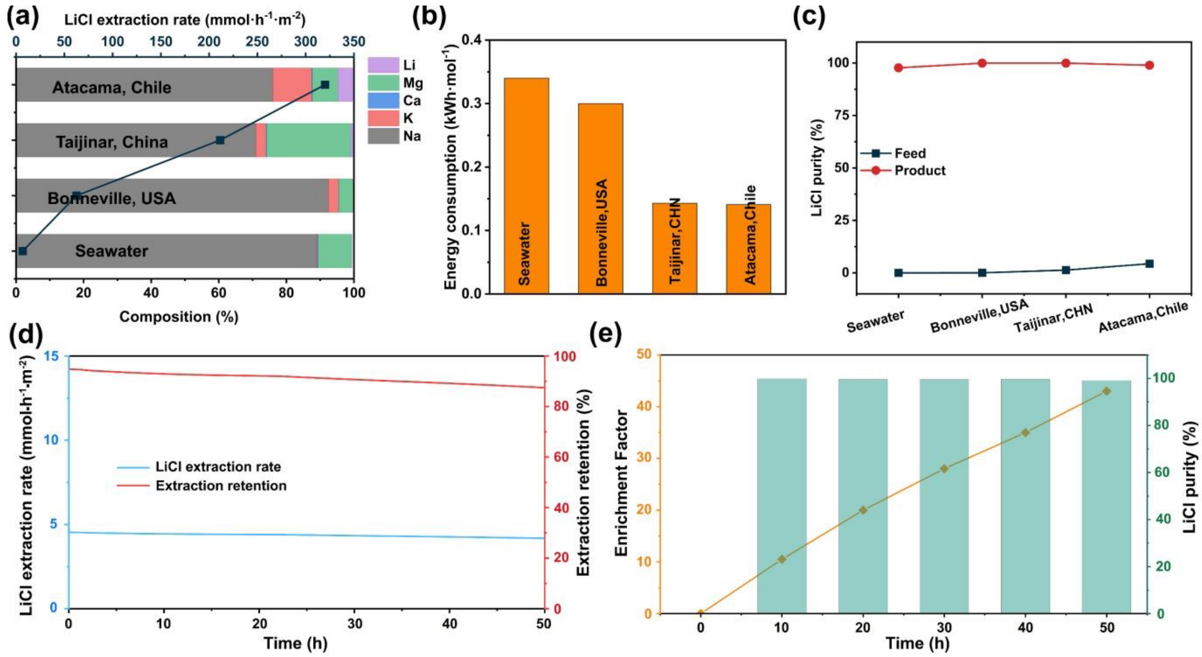

Figure 3. (a, b) Average rate and energy consumption for lithium extraction from simulated brines with different composition and 5 V rm-ED cell voltage, and (c) lithium purity in feed and product; (d) Lithium extraction rate and the rate retention as function of time with simulated seawater feed and 5 V rm-ED voltage, and (e) lithium enrichment factor and purity in the product.

6. The authors should perform cyclic voltammetry (CV) measurements on the ceramic membrane to confirm its electrochemical stability window and test its stability under high voltage conditions (e.g., over one week or longer).

Response: We sincerely appreciate the reviewer's valuable suggestion. We agree that ensuring the stability of the LSTH membrane in its working environment is crucial. However, since the LSTH membrane functions as a passive component in the rm-ED device and is not directly connected to the electrodes, its electrochemical stability potential window and the applied cell voltage are not directly relevant to its role in this setup.

Nevertheless, we recognize the importance of verifying the membrane's stability, particularly since it is exposed to an acidic environment throughout the extraction process (We used 0.01M HCl solution for the extraction stream to provide some initial ion conductivity and avoid introducing any metal cations). To address this, we tested the LSTH membrane under highly acidic conditions (0.1M HCl solution) for 14 days. The

results showed no significant changes in chemical composition or morphology (Figure S10), indicating excellent stability in acidic environments. Based on these results, we believe the LSTH membrane is stable enough for its operational conditions in the rm-ED system.

While we did not perform CV measurements, we believe that the membrane's performance is adequately supported by its demonstrated stability in the acidic extraction environment. However, we are open to conducting further tests, including CV measurements and high voltage tests, to explore its electrochemical stability window and stability under high voltage conditions if necessary for future studies.

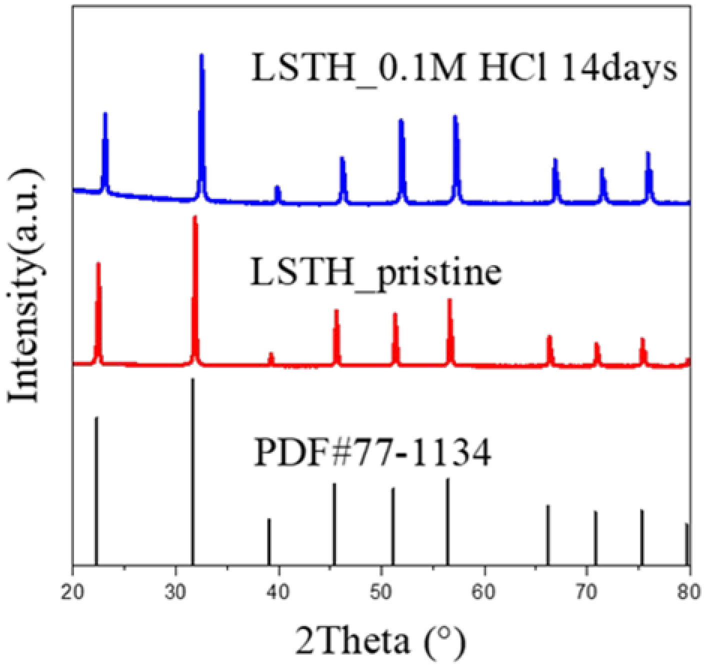

Figure S10. XRD data of LSTH before and after extended chemical stability test in an acidic environment.

7. The authors should measure the polarization curves of the Fe^{2+}/Fe^{3+} electrodes to determine the overpotential of the electrodes under different operating loads.

Response: We sincerely appreciate the reviewer's valuable suggestion and agree that a good knowledge of overpotential of the electrodes under different operating loads is critical for evaluating the system's performance. In response to the comment, we conducted polarization curve measurements of the Fe^{2+}/Fe^{3+} flow electrode solution by varying the potential. These measurements allowed us to determine the overpotentials of the electrodes at different operating currents. The data are presented below in form of Tafel plot (Figure R2), which indicate fast Fe^{2+}/Fe^{3+} redox kinetics and rapid current increase with overpotential. This is consistent with the voltage distribution measurements across the cell (see response to comment #8 below), which indicate an

overpotential of 0.68 V at the two electrodes with a 5 V applied cell voltage, accounting for just over 10% of the total voltage.

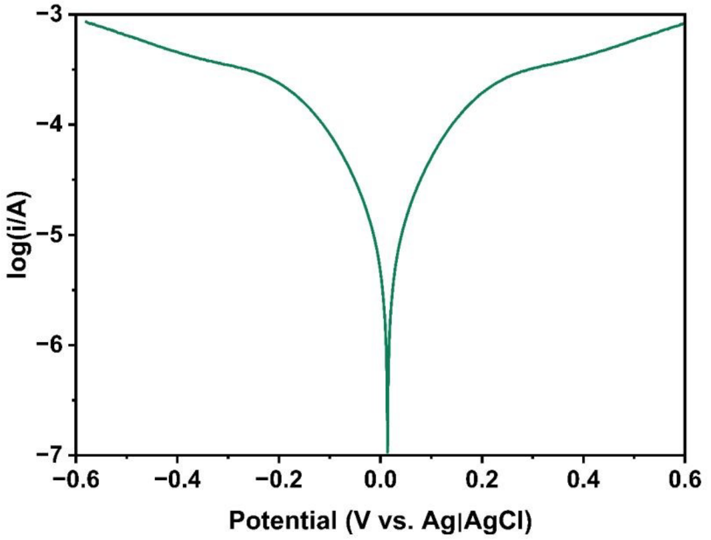

Figure R2. Tafel plot for Fe^{2+}/Fe^{3+} electrochemical redox process, obtained in an electrolyte containing 50 mM $FeCl_3$ and 50 mM $FeCl_2$ with a scan rate of 1 mV·s^{-1}.

8. The studied system is an aqueous system, and a voltage of 5 - 10 V is very high. I wonder how this is possible. The authors should measure the voltage distribution across the cell to determine how much of the applied 5-10V is consumed by the electrodes, the AEM, and the ceramic membrane, respectively.

Response: We sincerely appreciate the reviewer's valuable comment and agree that investigating the voltage distribution is crucial for assessing the risk of side reactions in the system. In response, we performed experiments to measure the voltage distribution across the different components of the rm-ED system.

For this, we inserted copper foils as connectors into each layer of the system and measured the voltage between different points using a multimeter while operating the cell at an applied voltage of 5 V. The results showed that the majority of the voltage (4.14 V) was used to drive lithium ions through the LSTH membrane. The measured voltages at the two electrodes, two AEMs, feed chamber, and product chamber were 0.34 V, 0.06 V, 0.04 V, and 0.02 V, respectively. Specifically, the overpotentials at the two electrodes summed to 0.68 V, which is well below the 1.23V thermodynamic threshold for water electrolysis side reactions (Figure R3). These findings suggest that even with an applied voltage of 5V, the likelihood of side reactions is minimal. The significant ohmic loss through the LSTH membrane, primarily due to its 1 mm thickness, accounted for most of the applied voltage. Reducing the membrane thickness would significantly improve energy efficiency.

Because the voltage distribution measurements are preliminary and require further investigation, we have not included this data in the revised manuscript.

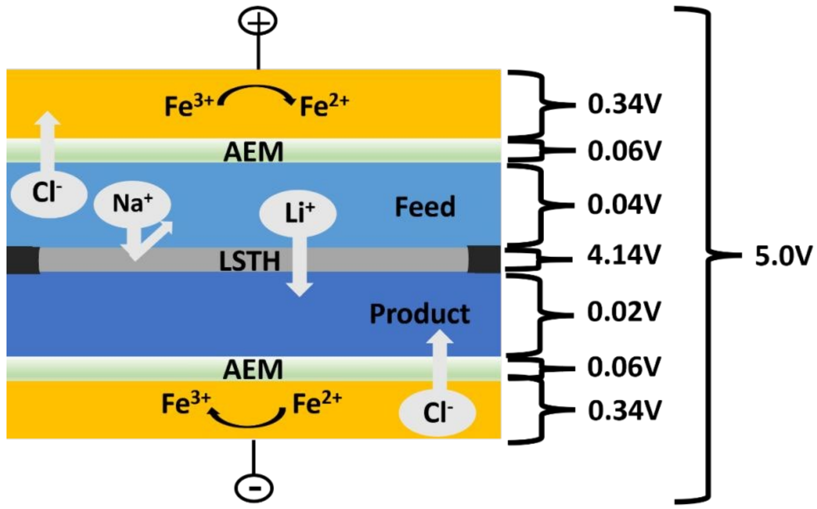

Figure R3. Voltage distribution across the rm-ED cell operating at 5 V.

Reviewer: 2

Recommendation: Reconsider after major revisions noted.

Comments:

This work designs a redox-mediated electrodialysis method to extract lithium from brines. They used a dense ceramic Li6/16Sr7/16Ta3/4Hf1/4O3 (LSTH) perovskite membrane, demonstrating high selectivity for lithium ions. Despite these advancements, several issues require further clarification:

(1). There appears to be a discrepancy in the lithium extraction rate and energy consumption data. Specifically, the manuscript states on page 3 (lines 28-31) that "…lithium extraction rate of 172 mmol·h-1·m-2 and an energy consumption of 0.102 kWh·mmol-1 were achieved when a 10 mM LiCl brine stream was fed to the RED cell"). Upon calculation, these figures seem contradictory given the supporting information provided in the "Lithium Extraction Tests" section (Figure S10), as detailed below:

As shown in the "Lithium Extraction Tests" section in supporting information file: the area of LSTH membrane was 0.5 cm2, the volume of product solution was 20 mL, the operation time was 0.5h (Figure S10) and the Li+ extraction rate (LER) and energy consumption (EC) were calculated by Eq. 1 and 2.

With the description shown in Page 3 in the manuscript and the data shown in Figure 2a and b, we can get the following calculations (The data provided by the authors are bolded):

(cLi+—cLi+,0) × Vproduct = LER × Ac × t =172 mmol·h-1·m-2 × 0.5 × 10-4 m2 × 0.5 h = 0.0043 mmol (By using Eq. 1)

V × C = EC × (cLi+—cLi+,0) × Vproduct = 0.102 kWh·mmol-1 × 0.0043 mmol = 0.0004386 kWh = 0.4386 Wh (By using Eq. 2)

The cell voltage was 5 V (Shown in lines 23-24, Page 3 in manuscript), so the value of the total charge transfer "C" is 0.08772 Ah. During the operation time 0.5 h, the average current should be 0.17544 A (Equal to 175.44 mA).

However, as shown in Figure S10, when the lithium concentration in the feed was 10 mM, the current was more than 0.2 mA but lower than 0.3 mA. These discrepancies require further explanation or correction.

Response: We sincerely appreciate the reviewer's valuable question and apologize for the two errors introduced in our calculations. First, we overcalculated the energy consumption by three orders of magnitude due to a unit conversion mistake, calculating in $kWh\cdot mmol^{-1}$ instead of the correct unit, $kWh\cdot mol^{-1}$. Additionally, we initially used a 20 mL volume for the product stream, but later reduced it to 10 mL in most experiments to increase the lithium concentration and obtain more precise ICP-OES measurements. Unfortunately, we failed to update this volume change in our calculations, leading to energy consumption values that were incorrectly reported as half of their actual values. These two errors together resulted in an overestimation of the energy consumption by

approximately 500 times. After carefully reviewing the raw data and recalculating several times, we have corrected these errors in the revised manuscript.

In the updated data, for example, for the experiment with 10mM LiCl feed and 5 V cell voltage, the total charge transfer can be calculated as follows:

$(C_{Li+} - C_{Li+,0}) \times V_{product}$ = LER × Ac × t =96 mmol·h^{-1}·m^{-2} × 0.5 × 10^{-4} m^{2} × 0.5 h = 0.0024 mmol (By using Eq. 1)

V × C = EC × $(C_{Li+} - C_{Li+,0}) \times V_{product}$ = 0.23 kWh·mol^{-1} × 0.0024 mmol = 0.00000055 kWh = 0.00055 Wh (By using Eq. 2)

The applied voltage is 5V, with an operation time of 0.5h. Therefore, the total charge transfer should be 0.00055 Wh/5 V = 0.00011 Ah, and the current should be 0.00011 Ah/0.5h = 0.000202 A = 0.202 mA. The average current measured for this experiment was 0.23 mA, closely matching the calculated value.

The revised data, along with the updated Figure 2, have been incorporated into the manuscript. Additionally, Table S2 has been added to the Supporting Information to provide a comprehensive summary of all experimental conditions and results. We have also attached an Excel spreadsheet detailing the raw data and calculations for full transparency.

(2). I recommend including tables in the supporting information file to detail the data used in Figure 2. These tables should list the concentrations of Li+ and Na+ in both the feed and recovery solutions before and after lithium extraction, applied voltage, and total charge transfer during the operation, to enhance data transparency and reproducibility.

Response: We sincerely appreciate the reviewer's valuable suggestion. We have added Table S2 to the Supporting Information to provide a comprehensive summary of all experimental conditions and results. We have also attached an Excel spreadsheet detailing the raw data and calculations for full transparency.

	**Applied Voltage(V)**	**Average Current (mA)**	**Feed**		**Product(10ml)**		**Charge transfer (C)**	**Energy Consumption (kWh·mol^{-1})**
			Li^{+}(mM)	Na^{+}(mM)	Li^{+}(mM)	Na^{+}(mM)		
10mM	5.0	0.23	10	/	0.24	/	0.41	0.23
50mM	5.0	0.33	50	/	0.51	/	0.60	0.17
100mM	5.0	0.39	100	/	0.63	/	0.71	0.16
150mM	5.0	0.41	150	/	0.69	/	0.74	0.15
200mM	5.0	0.45	200	/	0.80	/	0.81	0.14
0.5V	0.5	0.02	50	50	0.04	/	0.04	0.01
1.0V	1.0	0.07	50	50	0.13	0.001	0.13	0.03
3.0V	3.0	0.21	50	50	0.37	0.002	0.38	0.09
5.0V	5.0	0.34	50	50	0.51	0.002	0.60	0.17

Table S2. Experimental conditions and key performance results.

10.0V	10.0	0.57	50	50	0.72	0.007	1.02	0.40
1:1	5.0	0.33	50	50	0.54	0.009	0.60	0.16
1:5	5.0	0.32	50	250	0.49	0.012	0.58	0.17
1:10	5.0	0.33	50	500	0.50	0.006	0.60	0.16
1:50	5.0	0.33	50	2500	0.55	0.010	0.60	0.15
1:100	5.0	0.34	50	5000	0.53	0.015	0.61	0.17

(3). There is an inconsistency in the current profiles under identical conditions of lithium concentration and voltage in Figures S10 and S12. Figure S10 shows a smooth current curve (red line), whereas Figure S12 depicts an initial decrease followed by a stable current (green line). The reasons for these differences should be investigated and clarified.

Response: We sincerely appreciate the reviewer's valuable question. In response, we have repeated the relevant experiment and obtained more consistent data. The revised figure has been included in the supporting information as Figure S13.

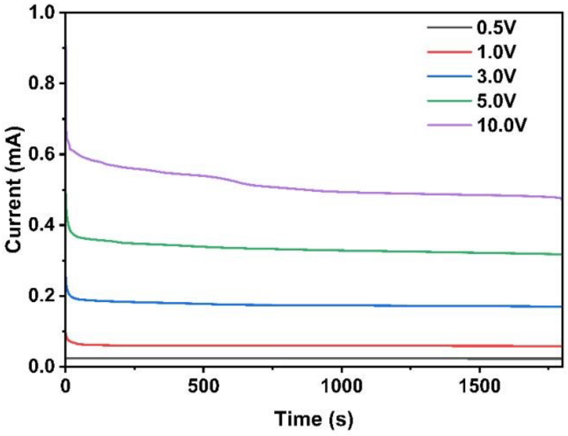

Figure S13. Measured rm-ED cell current as function of time, collected in lithium extraction experiments with 50 mM LiCl in the feed and different cell voltages.

The differences in the current profiles stem from two intertwined effects: the doublelayer phenomenon and the gradual balancing act between mass transfer and electrode reactions. When the voltage is first applied, particularly at 10V, a surge of capacitive charging takes place, causing the current to rise sharply. This current quickly diminishes as the capacitive effect subsides, typically within seconds. Simultaneously, the Fe^{2+}/Fe^{3+} redox species begin the experiment in uniform concentration throughout the electrolyte, but as the reactions proceed, they are gradually consumed near the electrode surface, creating a concentration gradient. This imbalance also contributes to the initial drop in current. As the system progresses, the mass transfer of Fe^{2+}/Fe^{3+} and their associated electrode reactions eventually reach a steady-state equilibrium, where the current stabilizes. This complex interplay between capacitive effects and redox reactions explains the initial dip seen in Figure S12 (green line), whereas Figure S10 (red line)

shows a smoother profile, likely due to faster stabilization or varied experimental conditions.

(4). The manuscript suggests that increased energy consumption is due to higher cell currents leading to greater ohmic losses and reduced charge efficiency, as seen in Figure S14. However, it is unclear why the charge efficiency (CE) at 0.5 V is lower than at 1 V. A more detailed analysis would be beneficial here to understand the impact of voltage on CE.

Response: We sincerely appreciate the reviewer's valuable suggestion and apologize for the earlier inaccuracy. The lower charge efficiency (CE) at 0.5 V, compared to 1 V, can be attributed to the very slow charge transfer and cation migration process occurring at such a low voltage (as indicated by a very low cell current). This introduces more significant measurement errors in ICP-OES quantitative analysis, leading to fluctuations in the calculated CE. We carefully repeated the experiments at 0.5 V several times and have determined the average CE at 0.5 V to be 95.1%. This value has been updated in Figure S15 in the revised Supporting Information.

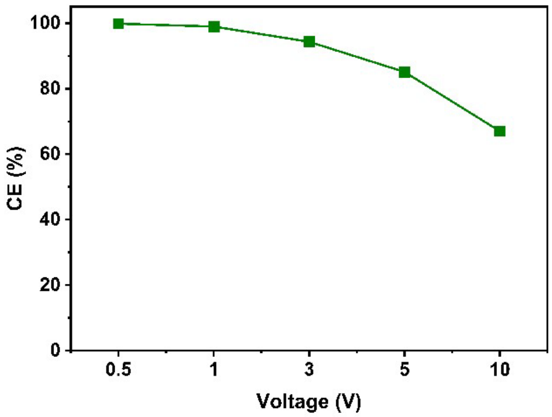

Figure S15. Determined charge efficiency in the lithium extraction experiments with 50mM LiCl and different cell voltages.

(5). There seems to be an error in Figure S18, where the operation times for seawater and Bonneville are shorter than those for Taijiner and Atacama. Please verify these data points and correct any potential inaccuracies.

Response: We appreciate the reviewer's attention to this detail and apologize for the inconsistency in our operation times. We have redone corresponding experiments and corrected the operation times for the seawater and Bonneville samples. Figure S18 has been updated in the revised Supporting Information.

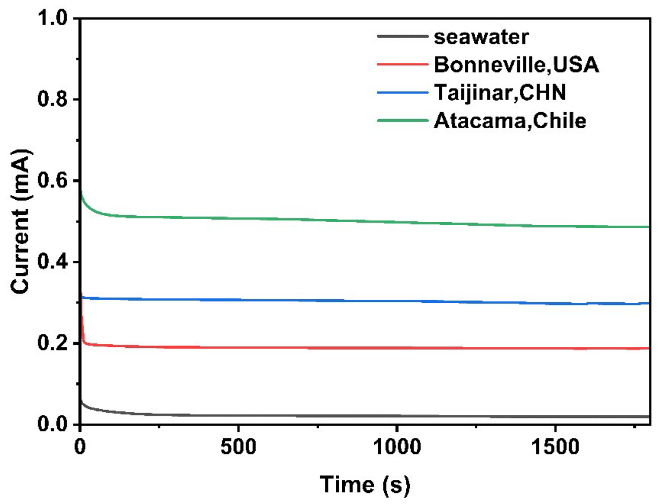

Figure S20. Measured rm-ED cell current as a function of time, collected in lithium extraction experiments with different simulated water sources as the feed and an applied voltage of 5 V.

(6). The ion concentration data from different brines cited in Table S2 rely solely on Reference 21. This citation might not provide sufficient validation for the data presented. A thorough review of the literature or additional experimental verification would strengthen the credibility of these values. And is "Aracama" spelled wrong? Is it "Atacama"?

Response: We sincerely appreciate the reviewer's valuable suggestion and relying on a single citation is insufficient to support the ion concentration data in Table S2. In response, we have revised Table S3 with additional citations from the literature to provide stronger validation for the data presented.

We also appreciate the reviewer for pointing out the typo. The name has been corrected to "Atacama, Chile" in the revised version.

Table S3. Concentrations of major cations in different water sources.

Brine source	Na^+(mM)	K^+(mM)	Ca^{2+}(mM)	Mg^{2+}(mM)	Li^+(mM)
Taijinar, China[5]	2449	112.5	4.9	831.1	44.6
Atacama, Chile[6]	3958	603.6	11.2	397	226.2
Bonneville, USA[7]	3610	127.9	1.4	164.6	8.14
Sea water averages[8, 9]	464.5	1.01	1.02	52.6	0.0243

(5) Sun, S.-Y.; Cai, L.-J.; Nie, X.-Y.; Song, X.; Yu, J.-G. Separation of magnesium and lithium from brine using a Desal nanofiltration membrane. *Journal of Water Process Engineering* **2015**, *7*, 210-217. DOI: https://doi.org/10.1016/j.jwpe.2015.06.012.

(6) Ogawa, Y.; Koibuchi, H.; Suto, K.; Inoue, C. Effects of the Chemical Compositions of Salars de Uyuni and Atacama Brines on Lithium Concentration during Evaporation. *Resource Geology* **2014**, *64* (2), 91-101. DOI: https://doi.org/10.1111/rge.12030.

(7) Yan, G.; Wang, M.; Hill, G. T.; Zou, S.; Liu, C. Defining the challenges of Li extraction with olivine host: The roles of competitor and spectator ions. *Proc Natl Acad Sci U S A* **2022**, *119* (31), e2200751119. DOI: 10.1073/pnas.2200751119 From NLM.

(8) Li, C.; Li, Z.; Wu, T.; Luo, Y.; Zhao, J.; Li, X.; Yang, W.; Chen, X. Metallogenic Characteristics and Formation Mechanism of Naomugeng Clay-Type Lithium Deposit in Central Inner Mongolia, China. *Minerals* **2021**, *11* (3), 238.

(9) Li, Z.; Li, C.; Liu, X.; Cao, L.; Li, P.; Wei, R.; Li, X.; Guo, D.; Huang, K.-W.; Lai, Z. Continuous electrical pumping membrane process for seawater lithium mining. *Energy & Environmental Science* **2021**, *14* (5), 3152-3159, 10.1039/D1EE00354B. DOI: 10.1039/D1EE00354B.

Reviewer: 3

Recommendation: Publish in ACS Central Science after minor revisions noted.

Comments:

Authors reported a redox-mediated (Fe^{3+}/Fe^{2+}) electrodialysis method to selectively extract Li from brines. By using a dense ceramic membrane, the authors showed a continuous operation system with good extraction rates, low energy consumption, and good Li selectivity. Thus, this work is believed to draw the interest of broad researchers and is suitable to be published in ACS Central Science. Before publishing, a few small aspects can be improved.

1. Utilizing electrodialysis with ceramic membranes to realize lithium extraction is not new. How good is this work compared to other works using electrodialysis (e.g. 1. Lithium Metal Extraction from Seawater. 2. A low-cost anodic catalyst of transition metal oxides for lithium extraction from seawater) and other methods, such as electrochemical intercalation, adsorption, solvent extraction, and ion exchange?

Response: We sincerely appreciate the reviewer's insightful question. As illustrated in Figure 4a, the key advantage of our rm-ED design lies in its theoretically infinite capacity for continuous operation without the consumption of chemicals or generation of side products. As long as there is a sufficient supply of brine, the system can continuously extract and enrich lithium in the product chamber. This sets it apart from other methods reported in the literature, which often require intermittent charge and discharge cycles, adding operational complexity.[10-14]

In comparison with other electrodialysis methods, such as those mentioned (e.g., Lithium Metal Extraction from Seawater, and the use of low-cost anodic catalysts of transition metal oxides), our rm-ED device offers several distinct advantages. Notably, our system achieves a high extraction rate of 320 $mmol \cdot h^{-1} \cdot m^{-2}$ and a remarkably low energy consumption of 0.014 $kWh \cdot mol^{-1}$, which are superior to many reported methods. This combination of continuous operation, higher extraction efficiency, and lower energy requirements enhances the practical applicability of our system. Furthermore, compared to alternative methods like electrochemical intercalation, adsorption, solvent extraction, and ion exchange, our rm-ED device stands out due to its scalability, operational simplicity, and energy efficiency. These improvements make our method not only more effective in lithium extraction but also more sustainable, reinforcing its advantages over existing technologies.[2-4]

2. People have reported electrodialysis method coupled with OER for lithium extraction (A low-cost anodic catalyst of transition metal oxides for lithium extraction from seawater). Could the authors provide some insightful comments on choosing redox pairs to complete the lithium extraction loop, in terms of efficiency, energy assumption, and environmental impact, cost, and scalability?

Response: We sincerely appreciate the reviewer's insightful question and agree that coupling electrodialysis with the oxygen evolution reaction (OER) presents a promising

approach for lithium extraction. However, there are several challenges that need to be addressed, challenges that are less pronounced when using iron-based redox couples.

Firstly, safety and environmental impact are significant concerns. Iron-based redox couples are generally non-toxic and environmentally benign, whereas transition metal oxide catalysts used in OER, depending on their composition, can pose environmental hazards and require careful handling. Additionally, transition metal oxide catalysts are prone to activity decay over time and face operational difficulties. In contrast, iron-based redox couples offer better stability and support continuous operation, mitigating these concerns.

In terms of energy efficiency, iron-based systems also offer advantages. Iron-based redox pairs typically exhibit lower redox potentials (for instance, 0.77 V vs. RHE for Fe^{2+}/Fe^{3+} while 1.23 V vs. RHE for OER) and require lower overpotentials (for instance, minimal onset overpotential for Fe^{2+}/Fe^{3+} while typically >200 mV onset overpotential for metal oxide-catalyzed OER), which translate into more efficient charge transfer and lower energy consumption. Transition metal oxide catalysts, while effective, may suffer from increased energy demands due to their inherent electrochemical properties.

Scalability is another important factor. Scaling an rm-ED system using iron-based redox couples is relatively straightforward—either by increasing the membrane size or adding more devices in parallel. This simplicity is a key advantage in large-scale lithium extraction applications.

Therefore, in our opinion, while OER-based electrodialysis systems have potential, ironbased redox couples offer distinct advantages in terms of safety, stability, energy efficiency, and scalability, making them a more practical choice for continuous, largescale lithium extraction.[13]

3. Figure 3d. the authors attributed the decrease in the extraction rate to the decrease of the lithium concentrations in the feed brine stream. But this could give a static dropping of the extraction rate. However, from Figure 3d, the extraction retention suddenly dropped in 20 – 30 h range. A better explanation should be provided.

Response: We sincerely appreciate the reviewer's valuable suggestion and agree that the sudden drop in extraction retention in the 20–30 hour range should not be solely related to gradual lithium concentration decrease in the feed brine stream. Upon further investigation, we believe this drop was likely caused by an unforeseen operational issue, such as a change in flow pattern due to a pump malfunction during long time operation or other operational disruptions.

To clarify this point, we repeated the experiment while carefully monitoring and minimizing the impact of any non-experimental factors. The revised Figure 3d with updated data, which show improved consistency in the extraction retention, have been incorporated into the revised manuscript. We believe these adjustments provide a more accurate reflection of the system's performance.

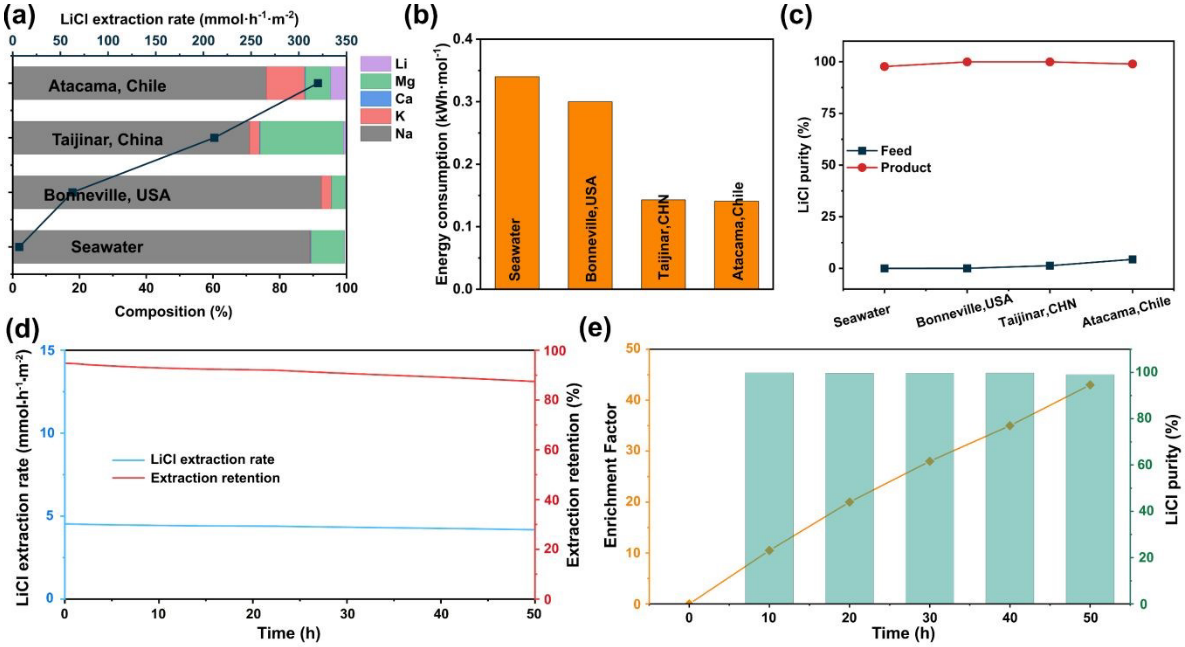

Figure 3. (a, b) Average rate and energy consumption for lithium extraction from simulated brines with different composition and 5 V rm-ED cell voltage, and (c) lithium purity in feed and product; (d) Lithium extraction rate and the rate retention as function of time with simulated seawater feed and 5 V rm-ED voltage, and (e) lithium enrichment factor and purity in the product.

References

(1) Xie, R.; Schrage, B. R.; Jiang, J.; Ziegler, C. J.; Peng, Z. Ferrocene Bis(Sulfonate) Salt as Redoxmer for Fast and Steady Redox Flow Desalination. *Molecules* **2024**, *29* (11), 2506.

(2) Song, Y.; Zhao, Z. Recovery of lithium from spent lithium-ion batteries using precipitation and electrodialysis techniques. *Separation and Purification Technology* **2018**, *206*, 335-342. DOI: https://doi.org/10.1016/j.seppur.2018.06.022.

(3) Ji, Z.-y.; Chen, Q.-b.; Yuan, J.-s.; Liu, J.; Zhao, Y.-y.; Feng, W.-x. Preliminary study on recovering lithium from high Mg2+/Li+ ratio brines by electrodialysis. *Separation and Purification Technology* **2017**, *172*, 168-177. DOI: https://doi.org/10.1016/j.seppur.2016.08.006.

(4) Ji, P.-Y.; Ji, Z.-Y.; Chen, Q.-B.; Liu, J.; Zhao, Y.-Y.; Wang, S.-Z.; Li, F.; Yuan, J.-S. Effect of coexisting ions on recovering lithium from high Mg2+/Li+ ratio brines by selective-electrodialysis. *Separation and Purification Technology* **2018**, *207*, 1-11. DOI: https://doi.org/10.1016/j.seppur.2018.06.012.

(5) Sun, S.-Y.; Cai, L.-J.; Nie, X.-Y.; Song, X.; Yu, J.-G. Separation of magnesium and lithium from brine using a Desal nanofiltration membrane. *Journal of Water Process Engineering* **2015**, *7*, 210-217. DOI: https://doi.org/10.1016/j.jwpe.2015.06.012.

(6) Ogawa, Y.; Koibuchi, H.; Suto, K.; Inoue, C. Effects of the Chemical Compositions of Salars de Uyuni and Atacama Brines on Lithium Concentration during Evaporation. *Resource Geology* **2014**, *64* (2), 91-101. DOI: https://doi.org/10.1111/rge.12030.

(7) Yan, G.; Wang, M.; Hill, G. T.; Zou, S.; Liu, C. Defining the challenges of Li extraction with olivine host: The roles of competitor and spectator ions. *Proc Natl Acad Sci U S A* **2022**, *119* (31), e2200751119. DOI: 10.1073/pnas.2200751119 From NLM.

(8) Li, C.; Li, Z.; Wu, T.; Luo, Y.; Zhao, J.; Li, X.; Yang, W.; Chen, X. Metallogenic Characteristics and Formation Mechanism of Naomugeng Clay-Type Lithium Deposit in Central Inner Mongolia, China. *Minerals* **2021**, *11* (3), 238.

(9) Li, Z.; Li, C.; Liu, X.; Cao, L.; Li, P.; Wei, R.; Li, X.; Guo, D.; Huang, K.-W.; Lai, Z. Continuous electrical pumping membrane process for seawater lithium mining. *Energy & Environmental Science* **2021**, *14* (5), 3152-3159, 10.1039/D1EE00354B. DOI: 10.1039/D1EE00354B.

(10) Li, Y.; Zhao, Y.; Wang, H.; Wang, M. The application of nanofiltration membrane for recovering lithium from salt lake brine. *Desalination* **2019**, *468*, 114081. DOI: https://doi.org/10.1016/j.desal.2019.114081.

(11) Chen, J.; Zhang, H.; Zeng, Z.; Gao, Y.; Liu, C.; Sun, X. Separation of lithium and transition metals from the leachate of spent lithium-ion battery by extraction-precipitation with p-tert-butylphenoxy acetic acid. *Hydrometallurgy* **2021**, *206*, 105768. DOI: https://doi.org/10.1016/j.hydromet.2021.105768.

(12) Shoghi, A.; Ghasemi, S.; Askari, M.; Khosravi, A.; Hasan-Zadeh, A.; Alamolhoda, A. A. Spinel H4Ti5O12 nanotubes for Li recovery from aqueous solutions:

Thermodynamics and kinetics study. *Journal of Environmental Chemical Engineering* **2021**, *9* (1), 104679. DOI: https://doi.org/10.1016/j.jece.2020.104679.

(13) Li, Z.; Chen, I.-C.; Cao, L.; Liu, X.; Huang, K.-W.; Lai, Z. Lithium extraction from brine through a decoupled and membrane-free electrochemical cell design. *Science* **2024**, *385* (6716), 1438-1444. DOI: doi:10.1126/science.adg8487.

(14) Yu, Y.; Yuan, Z.; Yu, Z.; Wang, C.; Zhong, X.; Wei, L.; Yao, Y.; Sui, X.; Han, D. S.; Chen, Y. Thermally assisted efficient electrochemical lithium extraction from simulated seawater. *Water Research* **2022**, *223*, 118969. DOI: https://doi.org/10.1016/j.watres.2022.118969.

oc-2024-013735.R2

Name: Peer Review Information for "Eco-friendly, Highly Selective Lithium Extraction by Redox-mediated Electrodialysis"

Second Round of Reviewer Comments

Reviewer: 3

Comments to the Author

The authors have addressed the reviewer's questions. It is recommended for publication.

Reviewer: 2

Comments to the Author

Generally, my comments have been well addressed. Now, it is suitable for publication in ACS Central Science.

Reviewer: 1

Comments to the Author

The authors have properly answered all the questions.

Author's Response to Peer Review Comments:

Dear Editor,

Thank you for the provisional acceptance of our manuscript “Eco-friendly, Highly Selective Lithium Extraction by Redox-mediated Electrodialysis” for publication in ACS Central Science. We sincerely thank the time and effort that you and all reviewers dedicated to providing valuable comments on our manuscript!

Following the instruction, we have made the formatting changes as follows:

1. We have added the email addresses of two corresponding authors on the first page of the manuscript and added label "Email".

2. We have added label "Synopsis".

3. We have added "TOC Graphic" above the TOC image on the last page of the manuscript.

Sincerely yours,

Zhenmeng Peng
